# Ecological determinants of altruism in prokaryote antivirus defense

**DOI:** 10.1186/s13062-025-00699-8

**Published:** 2025-12-20

**Authors:** Dmitry A. Biba, Kira S. Makarova, Yuri I. Wolf, Levi Waldron, Eugene V. Koonin, Nash D. Rochman

**Affiliations:** 1 Computational Biology Branch, Division of Intramural Research, National Library of Medicine, National Institutes of Health, Bethesda, MD, USA; 2 Oak Ridge Institute for Science and Education, Oak Ridge, TN, USA; 3 Institute for Implementation Science in Population Health, City University of New York School of Public Health, New York, NY, USA; 4 Department of Epidemiology and Biostatistics, City University of New York School of Public Health, New York, NY, USA; 5 Department CIBIO, University of Trento, Trento, Italy

## Abstract

Prokaryote evolution is driven in large part by the incessant arms race with viruses. Genomic investments in antivirus defense can be coarsely classified into two categories, immune systems that abrogate virus reproduction resulting in clearance, and programmed cell death (PCD) systems. Prokaryotic defense systems are enormously diverse, as revealed by an avalanche of recent discoveries, but the basic ecological determinants of defense strategy remain poorly understood. Through mathematical modeling of defense against lytic virus infection, we identify two principal determinants of optimal defense strategy and, through comparative genomics, we test this model by measuring the genomic investment into immunity vs. PCD among diverse bacteria and archaea. First, as viral pressure grows, immunity becomes the preferred defense strategy. Second, as host population size grows, PCD becomes the preferred strategy. We additionally predict that, although optimal strategy typically involves investment in both PCD and immunity, investment in immunity can also result in antagonism, increasing the likelihood that a PCD-competent cell will lyse due to infection. Together, these findings indicate that, generally, PCD is preferred at low multiplicity of infection (MOI) and immunity is preferred at high MOI. Finally, we demonstrate that PCD, which is typically considered to be an altruistic trait, is in some cases neutral and can be maintained in an unstructured population over an evolutionary timescale. Our work shows that the landscape of prokaryotic antivirus defense is substantially more complex than previously suspected.

**Supplementary Information:**

The online version contains supplementary material available at 10.1186/s13062-025-00699-8.

## Clinical trial number

Not applicable.

**Supplementary Information:**

The online version contains supplementary material available at 10.1186/s13062-025-00699-8.

## Introduction

Prokaryote evolution occurs amidst the perennial arms race with viruses [1, 2]. Established antiviral defense mechanisms are many and diverse, including restriction-modification systems (R-M) [3], toxin-antitoxin (TA) systems [4], CBASS [5], BREX [6], DISARM [7], CRISPR-Cas [8], and many more [9], with new defense systems being discovered at a high rate [10]. Most defense systems can be classified into two fundamentally distinct categories: immunity and programmed cell death, PCD (also often referred to as abortive infection). Both innate (e.g. R-M or BREX) and adaptive (CRISPR-Cas) immune systems are primarily focused on cell recovery through viral clearance. In contrast, PCD systems abolish the threat of virus transmission to neighboring cells by destroying infected cells before virus release. Thus, PCD is typically considered altruistic behavior, providing defense at the population level.

Whereas the phenomenology of PCD in prokaryotes is generally similar to that of the much better understood animal and plant PCD, the molecular machinery involved is only partially conserved. In metazoa, several distinct forms of PCD are recognized [11] with pathways centered on caspases, a distinct family of cysteine proteases, and STAND (Signal Transduction ATPases with Numerous Domains) NTPases playing a dominant role [12]. In plants, the best studied form of PCD is the hypersensitive response which also centers around a plant-specific subfamily of STAND NTPases known as NLRs, encoding both a nucleotide-binding site (NBS) and leucine-rich (LRR) repeat domains [13].

Although diverse caspase homologs, known as metacaspases, have been identified in bacteria and archaea [14–16], they do not seem to be the most common effectors of virus-induced PCD in prokaryotes. Notably, however, a caspase-like protease is a component of subtype III-E CRISPR systems, where it elicits PCD by generating a toxic cleavage product of another CRISPR-associated protein [17, 18]. Furthermore, bacterial STAND ATPases (denoted Antivirus STANDS, Avs) have been shown to elicit PCD upon recognizing specific viral proteins. The effectors involved, however, are typically nucleases rather than caspase-like proteases [19, 20]. Pyroptosis, a distinct form of PCD common in animals, is mediated by gasdermins, proteins that form membrane pores upon proteolytic activation [21]. Recently, gasdermin homologs found in some bacteria have been shown to cause PCD via a very similar mechanism [22, 23]. Thus, notably, PCD mechanisms that are rarely present in prokaryotes have taken center stage in eukaryotes. Conversely, the most common prokaryotic PCD devices, diverse Toxin-Antitoxin (TA) modules [24, 25], have not been inherited by eukaryotes, conceivably, due to the high toxicity of the respective toxins absent tight coregulation with the antitoxin that might be hard to achieve in eukaryotes due to the absence of operon organization.

The PCD and immune systems most commonly present in prokaryotes are Toxin-Antitoxin (TA) and Restriction-Modification (RM) systems, respectively. TA systems are two-gene modules, typically, with a long-lived toxin and a short-lived antitoxin [26]. Survival of TA-carrying organisms is predicated on the constant synthesis of the antitoxin, to accommodate for its fast decay. TA-mediated PCD is a passive mechanism that emerges from these kinetics. When the virus commandeers cellular synthetic machinery, antitoxin concentration drops, freeing the toxin and leading to cell damage or death. RM systems similarly contain at least two essential components, a site-specific methylase and a restriction endonuclease. The methylase modifies all recognition sites in the host genome whereas the restriction endonuclease cleaves that site if unmodified [27]. Following infection and the introduction of unmodified foreign DNA, the restriction endonuclease of the RM system cleaves the invading DNA, leading to clearance of the virus, and accordingly, representing a form of active immunity. Most other prokaryotic PCD and immune systems are built on the same general principles of regulated toxicity [9].

Most bacteria, and likely most archaea, possess a wide variety of both immune and PCD systems [28] which may be specific to a particular environmental stimulus [29] or virus(es) [30]. The interplay between immunity and PCD is complex, and not thoroughly understood. In addition to acting independently, PCD can be invoked as a second (and last) line of defense when immunity fails as exemplified by type III CRISPR systems (that combine the immune and PCD functions [31]) and the anticodon nuclease (ACNase) PrrC. PrrC persists as an inactive endoribonuclease in association with the RM complex, but is activated either by unmodified DNA or by the small phage-encoded anti-restriction peptide, causing translation inhibition by cleavage of the anticodon of tRNALys [32]. Recently, such activation of PCD factors resulting from inactivation of immune systems by phage-encoded antidefense proteins has been observed on a much broader scale, suggesting that this is a common outcome of the virus-host arms race [33, 34]. We are unaware of any examples of coordination in the reverse order, that is, immune activation following PCD failure. Uncovering the factors that shape the evolution and horizontal exchange of defense genes is central to the pursuit of general theoretical models of host-pathogen coevolution. In a more practical vein, understanding the ecological and host range of defense systems is required to evaluate candidates for phage therapy [35, 36].

In our previous work, we investigated, through mathematical modeling, the conditions in which PCD could emerge in coordination with multicellularity [37] and quantitatively characterized the evolution of defense strategies (immunity, PCD or both) for spatially structured bacterial populations [38]. It is generally believed that population structure (typically, spatial structure) is a necessary [39, 40] but not sufficient [41] condition for the evolution of PCD. This dependency stems from the altruistic nature of PCD which incurs a direct fitness cost and only an indirect fitness benefit, shared equally among all spatially neighboring individuals, irrespective of their genetic relatedness.

Here, we focus on ecological determinants of the optimal defense strategy against lytic viruses in a more fine-grained modeling framework and test these predictions through genomic and metagenomic data analysis. We proceed to evaluate the evolvability of the optimal strategy within a structured population using a Simpson’s paradox model framework [42, 43]. This model suggests that an altruistic trait can be maintained in a metapopulation of well-mixed subpopulations connected by weak migration even if the trait would not persist within an individual subpopulation. To our knowledge, this description fits well the ecology of gut and biofilm-forming bacteria. We find that, as expected, in the majority of ecologies studied, PCD fixation requires population structure. Surprisingly, however, under certain ecological conditions, we show that PCD is a neutral trait, and consequently, does not seem to be altruistic, persisting on an evolutionary timescale within well-mixed populations.

## Results

### A mathematical model of antivirus defense in prokaryotes via immunity or programmed cell death

**Fig. 1:**
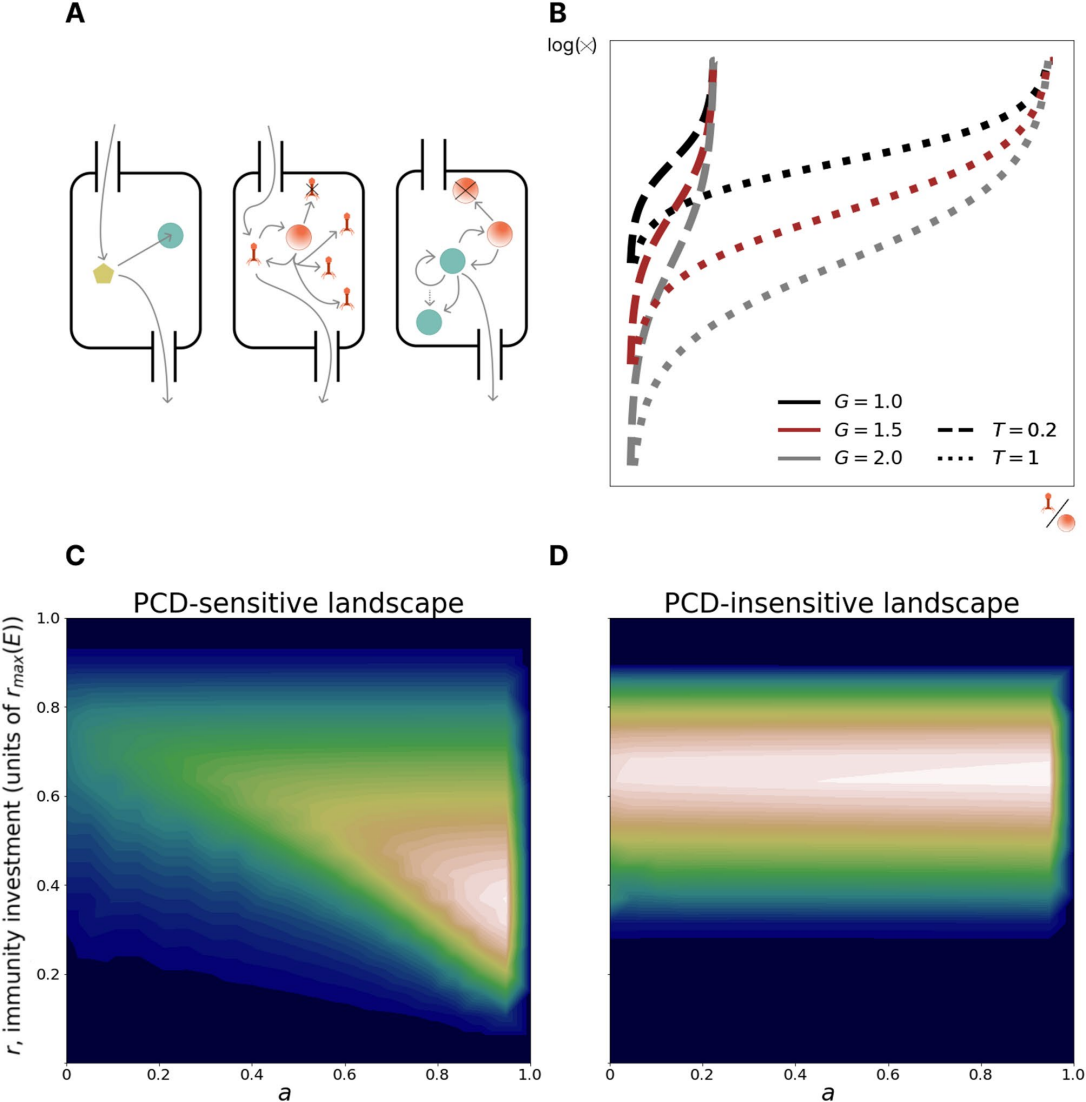
Model: interplay between immunity and PCD. **A**. Left: nutrient circulation in the chemostat. Center: virus circulation in the chemostat. Right: cell circulation in the chemostat. Unlike nutrient and virus circulation, cells are not present in influent media. Yellow pentagons represent nutrients; orange bacteriophages represent viruses; green and orange circles represent healthy and infected cells respectively. **B**. The log rate of cell lysis as a function of intracellular virus concentration. The model parameters G and T govern the lysis probability far from the lethal threshold and the lethal threshold, respectively. **C**. PCD-sensitive fitness landscape. x and y axes represent investments in PCD and immunity, respectively. Color reflects fitness measured by the size of the population with the given (a, r) phenotype (low fitness in blue, high fitness in white). The PCD-sensitive landscape has a pronounced peak (PCD-sensitivity = 0.94). **D**. PCD-insensitive landscape (PCD-sensitivity = 0.03). The legend is the same as in **С**. The peak of the PCD-insensitive landscape is not pronounced, instead there is a fitness plateau in the PCD axis at the optimal immunity value

Building on our recent work, where we explored the dichotomy between symmetric repair and asymmetric allocation of somatic damage during cell division [44], we present a mathematical model of a chemostat containing populations of prokaryotes, with an influx of lytic viruses. We model a chemostat of fixed, arbitrary volume diluted at rate $\:B$ with influent media of nutrient concentration $\:{\phi\:}_{0}$ and virus concentration $\:{\psi\:}_{0\:}$(Fig. 1A). Cell removal via dilution is considered extrinsic mortality. Cells consume media at rate $\:C$, depending on cell volume $\:p$, and ingest nutrients and are infected by viruses at a rate proportional to the concentration of each in the chemostat. Only cells devoid of viral particles are infected by viruses, that is, superinfection exclusion is enforced. Cells divide after reaching a critical size $\:2{p}_{0}$ (twice the birth size $\:{p}_{0}$). Viruses replicate within infected cells at rate $\:F$ resulting in cell lysis, at which point the intracellular virus enters the chemostat and becomes infectious, at a rate proportional to intracellular virus concentration. Lysis rate is determined by two parameters: $\:T$, specifying the maximum possible virus concentration (at which level the cell will lyse with probability 1) and $\:G$, specifying the average probability density for lysis at intermediate virus concentrations (Fig. 1B). The burst size distribution, that is, the number of virions produced at the time of cell lysis, depends on these parameters. The maximum absolute number of viruses within a cell is set at 1000, which covers the majority of reported viral burst sizes [45–48], and the concentration is normalized such that the smallest (newborn) cell containing 1000 viruses has a viral concentration of 1; therefore $\:0\le\:T\le\:1$.

Virus transmission can be prevented by an infected cell in two fundamentally distinct ways, by clearing virus via immunity or via PCD. Within our model, immune systems reduce the number of viruses inside the cell at a rate $\:r$ proportional to the cell volume. Investment in immunity comes at a growth cost $\:\frac{r}{E}$. PCD is modeled as obligatory self-destruction when virus concentration exceeds the threshold $\:1-a$. If $\:a=0$, PCD never occurs (burst probability is 1 at virus concentration 1) whereas if $\:a=1,$ PCD occurs as soon as a single virion enters the cell.

The above model yields the following system of equations for the dynamics of cells ($\:n$), nutrients ($\phi$) and viruses ($\:\psi\:$):

$$\eqalign{ & \:n(p,\:q,\:t + \Delta \:t)\: = n(p,\:q,\:t) + [A(1 - \frac{r}{E})*\phi \:* \cr & (n(p - 1,\:q,\:t)*(p - 1) - n(p,\:q,\:t)\:*\:p)\: + \cr} $$

$$\eqalign{ & \: + \:F*(n(p,\:q - 1,\:t)*(q - 1)\: - \:n(p,\:q,\:t)*q)\: \cr & + \:r\:*\:p\:*(n(p,\:q + 1,\:t)\: - \:n(p,\:q,\:t))\: - \cr}$$

$$\:-(B+(\frac{\rho\:}{T-\rho\:}{)}^{G}\left)*n\right(p,\:q,\:t\left)\right]\varDelta\:t$$

$$\eqalign{ & \:\phi \:(t + \Delta \:t)\: = \:\phi \:\left( t \right)\: + \:[(\phi {\:_0} - \phi \:)B - C\phi \cr & \:\:\sum {{\:_p}} \sum {{\:_q}} (p*n(p,\:q,\:t))]\Delta \:t \cr} $$

$$\eqalign{ & \:\psi \:(t + \Delta \:t)\: = \:\psi \:\left( t \right)\:\: + \:[(\psi {\:_0} - \psi \:) \cr & B - C\psi \:\sum {{\:_p}} (p*n(p,\:0,\:t)) \cr & + \sum {{\:_p}} \sum {{\:_q}} (q*n(p,\:q,\:t)*{(\frac{{\rho \:}}{{T - \rho \:}})^G})]\Delta \:t \cr}$$

See Methods for additional details.

Apart from discretization, this model has 8 degrees of freedom specifying ecological parameters. In this work, we exclusively study equilibrium behavior for which the timescale is arbitrary and we normalize the timescale by the growth rate, $\:A,$ to measure time in units of generations under the condition of maximum nutrient concentration. We ranged dilution rate, $\:B$, between 5% and 20% of the entire chemostat volume per generation, roughly corresponding to typical conditions for experimental cultures of *Escherichia coli* in a chemostat [49–51]. We modeled the behavior of the system with respect to variation in the remaining 6 ecological degrees of freedom evaluated over the entire range of $\:B$. For each possible ecological regime (specified by the parameter set {C, E,G, T,$\:{\phi\:}_{0}$,$\:{\psi\:}_{0}$}), we explored the fitness landscape with respect to the strategy parameters $\:a$ and $\:r$, reporting scaled investment in immunity $\:r$ normalized by the parameter $\:E$ such that, when $\:r/E$ approaches 1, the cell growth rate approaches 0.

For each ecological regime, the primary model output is the fitness landscape. This landscape is two-dimensional, parametrized over the 2 strategy parameters, investment in immunity and PCD. The first observation to come out of our model is that all fitness landscapes can be classified into two categories: those that have a single pronounced peak (Fig. 1C) and those that exhibit a broad plateau (Fig. 1D). In every case, this plateau is observed to run almost exactly parallel to the PCD investment axis. Consequently, while fitness is always sensitive to investment in immunity, such that minor deviations from the global optimum results in a sharp drop in fitness, landscapes can be either PCD-sensitive or PCD-insensitive.

We quantify PCD-sensitivity via the measure $\:1-\frac{n(a=0,\:r={r}_{opt})}{n=(a={a}_{opt},\:r={r}_{opt})}$ and label peaked landscapes that exceed a threshold of 0.1 as PCD-sensitive. The PCD-sensitive landscapes occupy about 37% of the explored space of ecological conditions, demonstrating that optimization of PCD investment is important in a wide variety of conditions, namely, in those with high viral replication rate, high carrying capacity, and low viral pressure (Supplementary Fig. 1). We note that $\:{\psi\:}_{0}$ is the extrinsic viral pressure. The viral concentration within the chemostat, $\:\psi\:$, is time dependent and the value of $\:\psi\:$ at pseudo-steady state can be higher or lower than $\:{\psi\:}_{0}$.

We additionally explored the dependence of the optimal strategy on burst size-mediating parameters $\:T$ and $\:G$ (Supplementary Fig. 2). Increasing $\:G$ (which decreases the lysis probability far from the lethal threshold) resulted in a fitness landscape with a plateau spanning intermediate PCD investments bordered by cliffs at both extremes of the PCD domain. When the burst size distribution is narrow, PCD remains effective at preventing virus transmission even when the virion concentration is high. In the limiting, idealized case where all cells burst at the mean value, setting the PCD threshold to be just below this value is 100% effective. Increasing $\:T$ (which increases the mean burst size) lowers optimal PCD investment, and also decreases optimal investment in immunity, a consequence of the increase of the average time to lysis.

Table 1 summarizes the range of parameters explored and identifies regions of the parameter space corresponding to PCD-sensitivity.

**Table 1:** Model parameters ranges and their effect on the PCD-sensitivity of the fitness landscape

Parameter	Description	Range	Increased PCD-sensitivity when parameter is high/low:
B	Chemostat dilution rate	0.05–0.2	High
C	Rate of nutrient/virus consumption	10^− 7^ − 10^− 5^	Low
$\:{\psi\:}_{0}$	Virus input rate	10^7^ − 10^12^	Low
E	Maximum immunity investment	0.0125–0.05	Low
F	Virus replication rate	0.8–8.0	High
G	Curvature of cell lysis rate function	1.0–2.0	Low
T	Maximum virus concentration	0.2–1.0	High

PCD-sensitive landscapes are ubiquitous with respect to any individual parameter: for any value of any parameter, there are values of the remaining parameters that correspond to a PCD-sensitive landscape. The representation of PCD-sensitivity is also nonuniform across each individual parameter range and here we report whether increasing or decreasing each parameter leads to overrepresentation of PCD-sensitive landscapes (for more details, see Supplementary Fig. 1)

### Testing the model predictions through genome analysis

To test our model predictions with empirical data on prokaryote antivirus defense investment, we analyzed 5121 species of bacteria and archaea with at least one representative complete genome from the intersection of the PADLOC [52] and Prok2311 [53] datasets (see Methods). For each genome, we identified all genes associated with PCD and immunity based on the published information on the functionality of prokaryotic defense systems [9, 54, 55]. Briefly, all defense systems capable of specific recognition and targeting of foreign molecules (typically, nucleic acids), such as CRISPR-Cas, Restriction-Modification, BREX, DISARM, DNA phosphorothioation, and others, were classified as immunity, whereas systems that cause dormancy or cell death such as various abortive infection modules, toxin-antitoxins, CBASS, and others, were classified as PCD. The complete list of the immunity and PCD assignments is provided in Supplementary Table 1. The genomic investment was then estimated as the fraction of the genome length (in nucleotides) occupied by each type of defense system. This approach accounts for both transcriptional and translational costs of investment in defense relative to the total reproduction burden of the organism (proportional to genome size). See Methods for details.

Another key parameter in our model, population size, cannot be reliably estimated from genomic sequence data alone. Note that, throughout this manuscript, we refer to population size as “census” population size to distinguish this measure from the effective population size (*Ne*). The census population size refers to the total number of individuals within the given group and not the total number of individuals within the species. To investigate the effect of population size on the optimal defense strategy, we focused on bacteria living in the human gut. Working with rank abundances taken from curatedMetagenomicData [56], we classified species into two groups, those that are consistently found in gut microbiomes at high and low abundance. Species for which the 1st quartile of the rank abundance distribution across samples fell above the threshold $\:{T}_{a}$ were considered high-abundant; those for which the 3rd quartile of the same distribution fell below this threshold were considered low-abundant (see Methods for more details). We then identified representative genomes for these species in the dataset described above.

### PCD investment decreases with viral pressure

We first explored how the optimal defense investment strategy depends on the viral pressure - the rate at which cells encounter viruses. In the model, viral pressure is determined by $\:{\psi\:}_{0}$, virus concentration in the influent media. The viral pressure is measured by the percentage of the genome dedicated to antivirus defense. Although the pressure from lytic viruses is not explicitly imprinted in the genomes, we considered the total investment in defense as a proxy for viral pressure, the rationale being that environments with higher viral load create selective pressures for increased investment in defense. This assumption has been explicitly validated across diverse microbial ecologies for the prevalence of CRISPR-Cas systems [57], and more recently, for a variety of other defense systems [58].

**Fig. 2:**
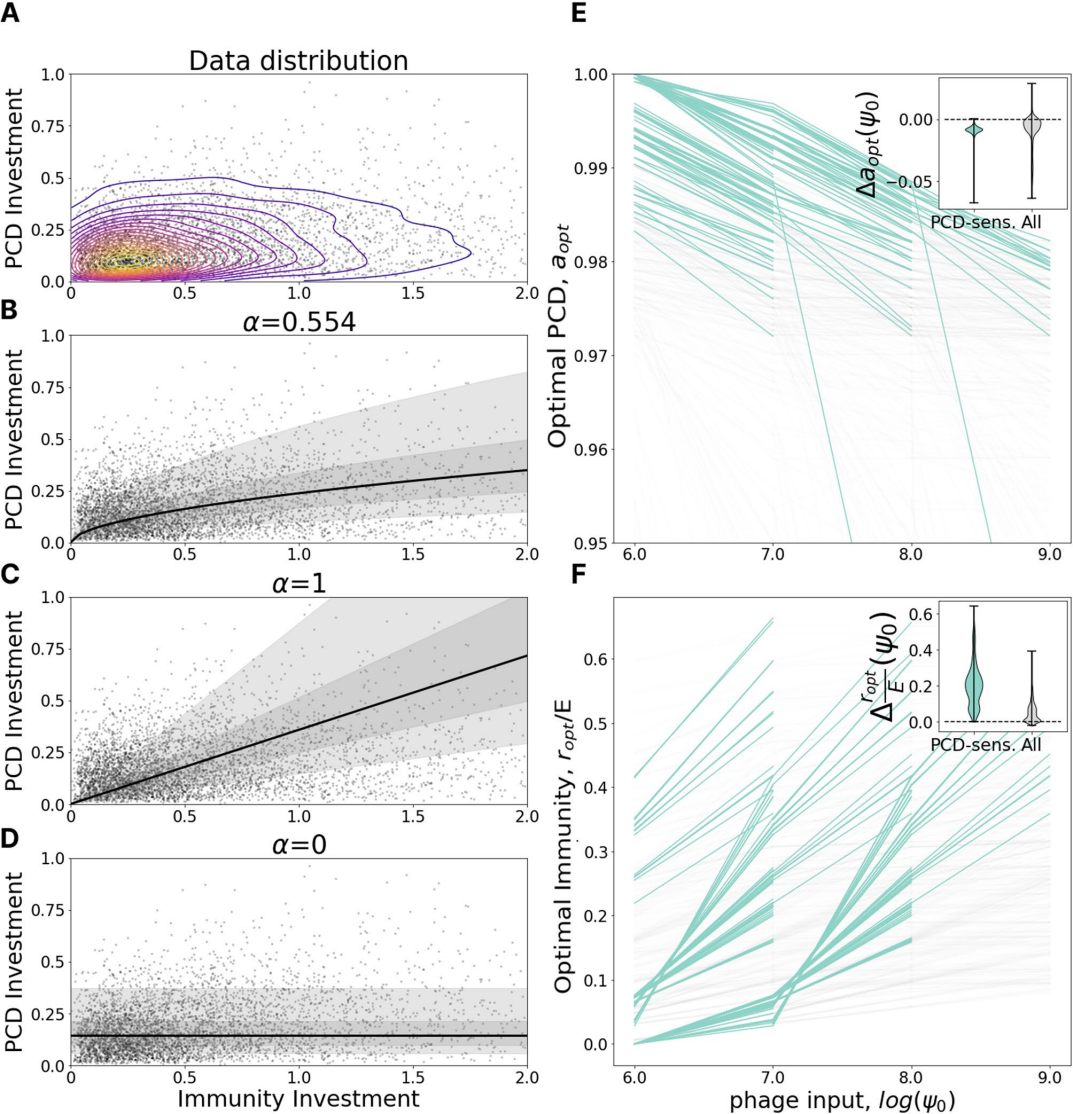
PCD investment decreases with viral pressure. **A.** Genomic investment in PCD vs. immunity among 5121 prokaryotic species. Contours indicate a gaussian KDE with 20 levels uniformly distributed between the minimum and maximum density value. **B-D.** Fit of $\:{I}_{PCD}\:=\:\beta\:*{{I}_{Imm}}^{\alpha\:}$ with $\:\alpha\:=0.554$ (Maximum Likelihood), 1 and 0. Bayesian information criterion with $\:\alpha\:=$1, 0 is 1247 and 3735 higher than ML respectively, indicating strong preference for the Maximum Likelihood model. The region bounding 5%-95% of the probability density is shaded in light gray, 25–75% in dark gray. **E.** Dependence of optimal PCD investment on viral pressure, $\:{\psi\:}_{0}$. Each line represents a fixed environment with varied $\:{\psi\:}_{0};\:$pairs of neighboring environments with both PCD-sensitivities > = 0.1 are highlighted in teal. The inset shows the distribution of differences in optimal PCD investment between neighboring points within the same line ($\:{\psi\:}_{0}$-neighboring environments). **F.** Same as in **E** for optimal investment in immunity relative to the maximum possible immunity investment $\:E$

Within the model, we tracked the change in optimal investment in PCD and immunity (the location of the peak on the fitness landscape) varying the viral pressure $\:{\psi\:}_{0}$ and holding all other parameters constant. Model predictions indicate that, as viral pressure increases, the optimal PCD investment, $\:a$, decreases (Fig. 2E) whereas the optimal immunity investment, $\:r/E,$ increases (Fig. 2F). Note that the plateau of PCD-insensitive landscapes makes the identification of the global optimum relatively less precise.

To test this prediction, we fit a power law regression $\:{I}_{PCD}\:=\:\beta\:*{{I}_{Imm}}^{\alpha\:}$ to the genomic data where $\:{I}_{PCD}\:$ and $\:{I}_{Imm}\:$are investments in PCD and immunity, respectively, and $\:\alpha\:$ and $\:\beta\:$ are free parameters. The noise in the regression is distributed lognormally with standard deviation $\:\gamma\:$ for the underlying normal distribution. Parameter $\:\alpha\:$ specifies the relative rate at which organisms tend to invest in PCD in comparison to immunity as total investment in antivirus defense increases. Using a maximum likelihood procedure (see Supplementary Methods), we identified the best fit $\:\alpha\:$= 0.554 (Fig. 2B) to represent a sublinear dependence. Under this sublinear dependence, as total investment in defense (proxy for viral pressure) increases, the contribution of PCD relative to immunity declines. We also compared the fit to “naive” linear, $\:\alpha\:=1$ (Fig. 2C) and flat, $\:\alpha\:=0$, models (Fig. 2D). The linear dependence corresponds to equal investment in immunity and PCD, independent of the total defense investment, whereas the flat dependence corresponds to investment in PCD that is constant and independent of the total defense investment (consequently, species which invest more in defense, in total, invest relatively more in immunity and less in PCD). Bayesian information criterion with $\:\alpha\:=$1, 0 is 1247 and 3735 higher than ML, respectively, indicating strong preference for the sublinear ML model, rejecting both naive dependencies, and supporting the model prediction.

### PCD investment increases with population size

**Fig. 3:**
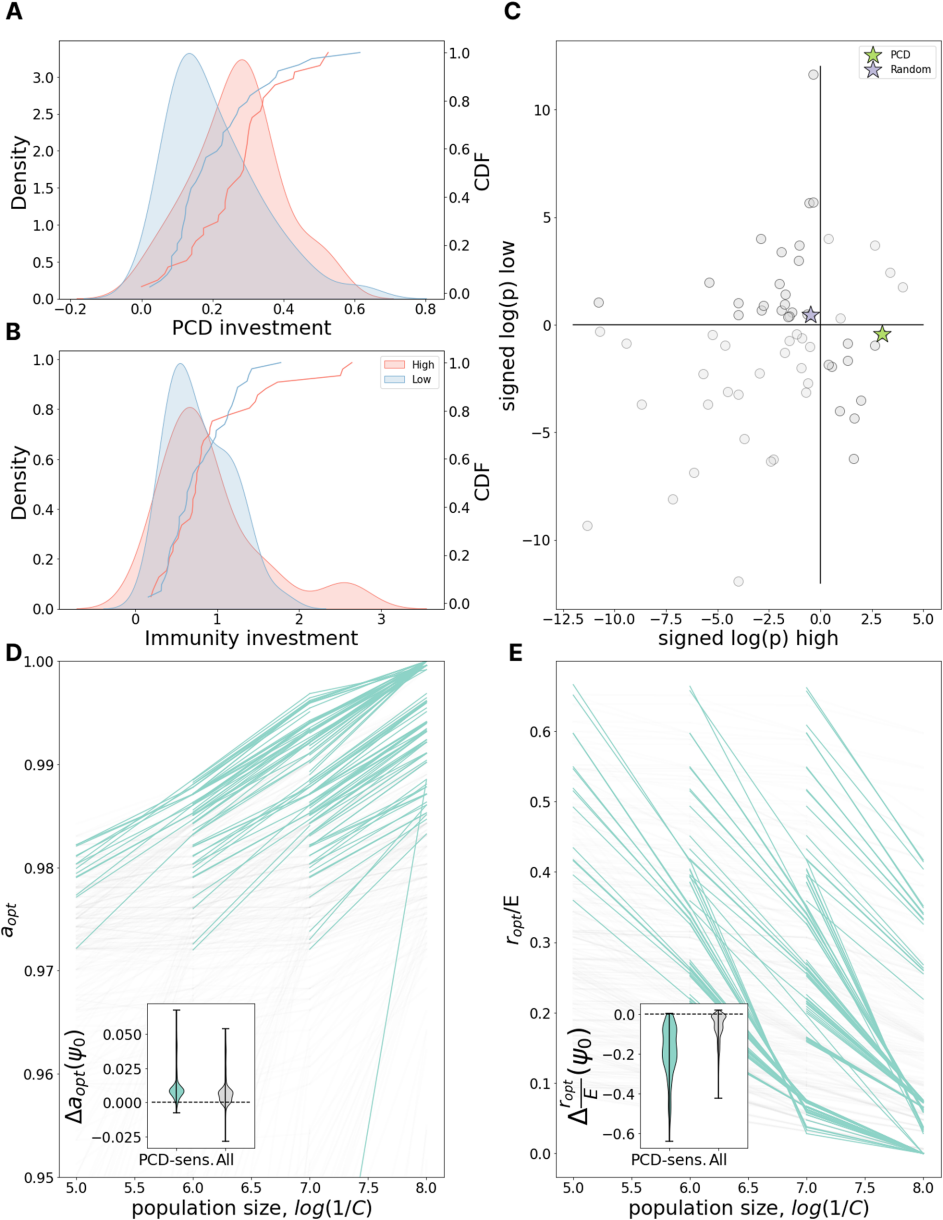
PCD investment increases with population size. **(A)** The distribution of PCD investment in high- (red) and low- (blue) abundant species and its gaussian KDE approximation. **(B)** Same as in **A** but for Immunity investment. **(C)** x and y axes represent signed logarithms of p-value for the deviation of investment in different pathways from genome size expectation for high-abundant and low-abundant species respectively (-log(p) for positive deviations, log(p) for negative deviations). Blue star represents investment in PCD, green star - in a set of random proteins. Quadrants from top-right moving counterclockwise are labeled as high-high, low-high, low-low, and low-high in the text. **(D)** Dependence of optimal PCD investment on carrying capacity of the environment, $\:1/C$. Each line represents a fixed environment with varied $\:C;\:$pairs of neighboring environments with both PCD-sensitivities > = 10% are highlighted in teal. The inset shows the distribution of differences in optimal PCD investment between neighboring points within the same line ($\:C$-neighboring environments). **(E)** Same as in **D** but for optimal investment in immunity relative to the maximum possible immunity investment $\:E$. **D-E.** *a*_*opt*_ and *r*_*opt*_ specify optimal investment in PCD and immunity for the environment, respectively. Environmental parameter *ψ*_*0*_ specifies the extrinsic viral pressure

We proceeded to explore how the census population size impacts the antivirus defense strategy. In the model, the population size is known. In the data, the relative census population sizes are estimated via relative metagenome rank abundance. We divided the data into two groups of species, those consistently found to be among the most highly abundant in the human gut and those which are consistently observed across individuals at low abundance (Fig. 3).

In our chemostat model, the population size depends on several parameters. The principal determinant is the nutrient/phage acquisition rate $\:C$ which regulates the maximum possible population size, that is, the carrying capacity of the environment. $\:C$ is inversely proportional to the carrying capacity and as $\:1/C$ grows, investment in PCD increases (Fig. 3D) while investment in immunity drops (Fig. 3E). Critically, however, decreasing the cost of immunity by increasing $\:E$, reduces the dependence on $\:1/C$ for immunity but not for PCD (Supplementary Fig. 3). It follows that optimal PCD and immunity investments do not strictly covary.

To test these predictions, we evaluated the average PCD investment among low- and high-abundant species. In agreement with the model, we found that, on average, highly abundant species invest more in PCD than low-abundant species (Fig. 3A, Mann-Whitney test *p* = 0.0188). In contrast, immunity investment does not differ between the two groups (Fig. 3B, Mann-Whitney test *p* = 0.655). This finding is also in agreement with the model assuming $\:E$ is large and suggests that in the human gut microenvironment, investment in immunity could be relatively less costly than in other ecological niches.

However, unlike the analysis in the preceding section, in which the genome length distribution among species with above average investment in immunity did not significantly differ from that for species with below average investment in immunity, species that are highly abundant in the human gut have substantially larger genomes than those of low abundance (Supplementary Fig. 4). To account for the systematic bias introduced by this difference in genome size, we performed additional analysis assessing the deviation from the expectation for genomic investment in PCD given the genome size alone. This analysis was repeated for genomic investment in a wide variety of other functional systems as well as a random collection of genes (see Methods).

Each of the 63 systems (PCD, random gene collection, and 61 COG pathways) can be assigned to one of 4 categories based on whether that system was observed to be over- or underinvested compared to genome size expectation in high- and low-abundant species (Fig. 3C). We identified 25 systems to be underinvested in all gut species, independent of their relative abundance (low-low). The acquisition and maintenance of these systems may be subject to relatively weaker selection pressures within the human gut through mechanisms which are unrelated to their census population size. Only 5 systems were identified to be overinvested in all gut species (high-high), potentially reflecting the relative “richness” of the human gut in contrast to other microenvironments. For 33 systems, the deviation from expectation was of opposite signs for high- and low-abundant species, indicating that evolution of these systems may be impacted by census population size within this microenvironment. We identified 22 systems which were overinvested among low-abundant species (high-low) and only 11 overinvested in high-abundant species (low-high). PCD belongs to the latter category and stands out as the system with the lowest p-value, whereas the investment in random proteins is well predicted by genome size alone. Moreover, among systems overrepresented in high- and underrepresented in low-abundant species, PCD also showed one of the most pronounced deviations from the expectation for the difference between high- and low-abundant species (Supplementary Fig. 5). Overall, supporting model predictions, the data strongly suggest that bacterial species with large population sizes within the human gut invest more in PCD than species which, while also ubiquitous in this environment, are present in lower numbers. More work is required to evaluate causality (directionality).

### Antagonism between investments in PCD and immunity

In the preceding sections, we demonstrated that the relative genomic investment in PCD vs. immunity decreases with increasing viral pressure and increases with increasing population size. These observations from genome analysis correspond well to our model predictions for the optimal defense investment strategy, motivating exploration of intracellular viral dynamics within the model which could direct future experimental efforts. Building on our previous work which showed that the growth law governing intracellular somatic damage accumulation, independent of the average rate of accumulation, is the primary determinant of the optimal damage control strategy [44], we explored the dependence of optimal defense strategy on the virus replication rate $\:F$. For this analysis, it is useful to consider multiplicity of infection (MOI). Within our model, MOI of an idealized population at carrying capacity is specified by the product $\:C{\psi\:}_{0}$ (recall that $\:{\psi\:}_{0}$ specifies virus influx, while $\:1/C$ is proportional to the carrying capacity). It should be emphasized that $\:{\psi\:}_{0}$ is the extrinsic viral pressure and, alternatively, one may define MOI to be $\:C\psi\:$, a time-dependent quantity reflecting the current chemostat viral concentration. To avoid confusion, below, we refer to $\:C{\psi\:}_{0}$ as “extrinsic MOI”.

**Fig. 4:**
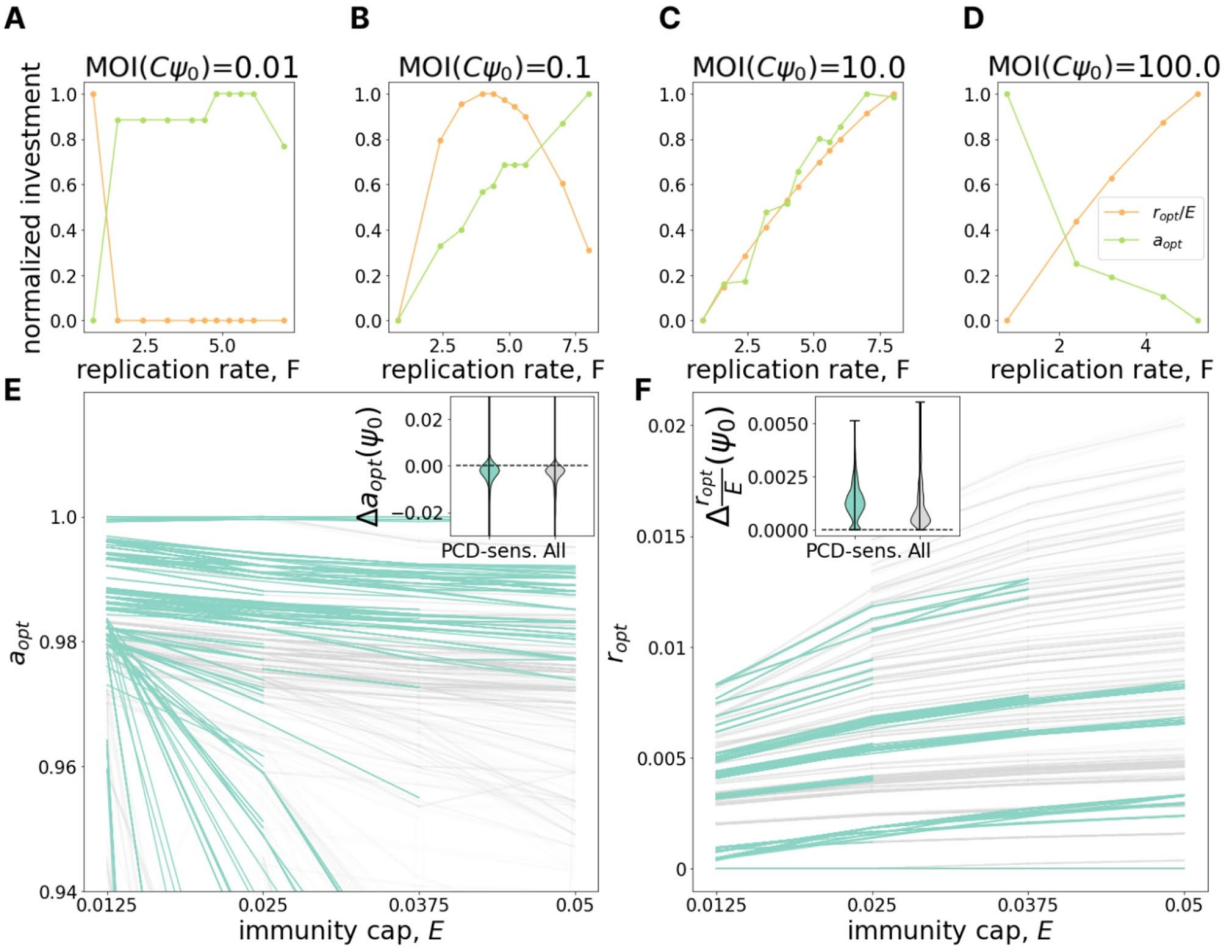
Antagonism between immunity and PCD. **A-D.** The change of optimal investment in PCD (green) and immunity (orange) with increasing virus replication rate $\:F$ (x-axis) for different values of extrinsic MOI ($\:C{\psi\:}_{0}=\{0.01,\:0.1,\:1,\:100\}$). Model parameters *C* and *ψ*_*0*_ specify cell media uptake rate and extrinsic viral pressure, respectively. Investments in PCD and immunity are scaled by the maximum investment in PCD and immunity within the panel respectively. **E.** Dependence of optimal PCD investment on maximum immunity, $\:E$ (immunity cap). Each line represents a fixed environment with varied $\:E;\:$pairs of neighboring environments with both PCD-sensitivities > = 10% are highlighted in teal. The inset shows the distribution of differences in optimal PCD investment between neighboring points within the same line ($\:E$-neighboring environments). **F.** Same as in **E** but for optimal investment in immunity (note the absence of the usual scaling by the maximum possible immunity investment $\:E$). **E-F.** *a*_*opt*_ and *r*_*opt*_ specify optimal investment in PCD and immunity for the environment, respectively

Figures 4A-D show that the relationship between investment in immunity and PCD can be antagonistic. At low extrinsic MOI (Fig. 4A), as viral replication rate grows, optimal investment in PCD increases whereas optimal investment in immunity decreases. The relationship is reversed for high extrinsic MOI (Fig. 4D). For intermediate extrinsic MOI, the optimal immunity investment can be non-monotonic with respect to viral replication rate (Fig. 4B). These results can be interpreted as follows. Increasing investment in immunity reduces the effective viral replication rate leading to two opposing effects. In some cells the virus is completely cleared, reducing transmission. In other cells, the virus is not cleared and now “slips under the PCD radar”, increasing transmission. This second effect clearly demonstrates the potential for antagonism between the two defense strategies, whereby investment in immunity decreases PCD efficacy. Examining the change in optimal PCD investment with respect to decreasing the cost of immunity investment (increasing E) further clarifies these dynamics. As expected, immunity cost reduction leads to an increase in optimal immunity investment (Fig. 4F), but unexpectedly, this cost reduction also decreases the optimal investment in PCD (Fig. 4E), reinforcing the notion of antagonism. We emphasize that, for such an antagonistic relationship to exist, the immune system must not be completely effective. In the limiting case of a pseudo-lysogenic state, infected cells must continue to produce at least 2 virions on average.

### PCD fixation dynamics in structured populations

The modeling approach introduced in the preceding sections addresses the ecological determinants of the theoretical optimal PCD investment but not the conditions required for the emergence or persistence of PCD during evolution. PCD is unlikely to emerge or persist in PCD-insensitive ecologies where, even in the absence of competition, PCD-competency confers a negligible fitness advantage. Even in PCD-sensitive ecologies, competition with subpopulations that never commit PCD presents an obstacle to evolutionary stability. PCD is typically considered to be an altruistic trait as committing PCD incurs a direct fitness cost and only an indirect fitness benefit, shared equally among all spatially neighboring individuals, irrespective of their genetic relatedness. Within a single well-mixed population, such as our chemostat model, the emergence through mutation or introduction through migration of a “cheater” genotype, which never commits PCD, would be expected to outcompete the PCD-competent subpopulation, driving it to local extinction.

**Fig. 5:**
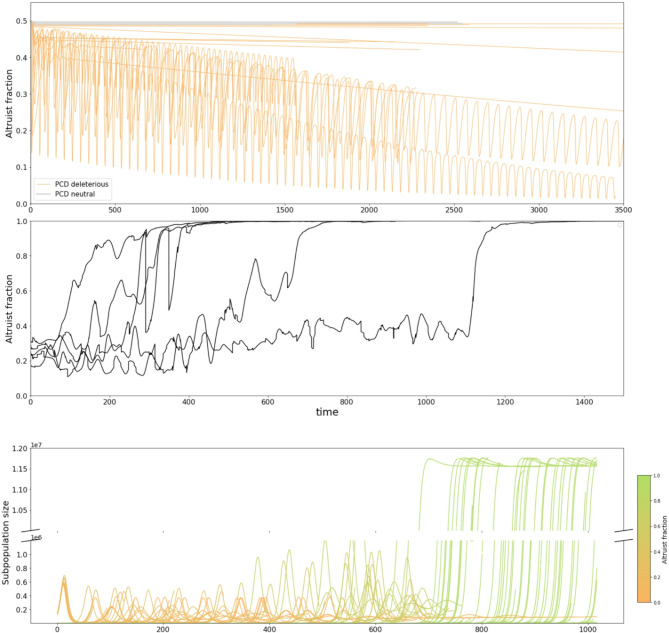
PCD fixation in structured populations. **(A)** Traces of altruist (PCD-competent) fraction for 20 simulations of altruist-cheater competition. All 20 ecologies are PCD-sensitive. Simulations were evaluated for an established interval and not until fixation. See Supplementary Fig. 7 for a representative simulation evaluated until fixation. **(B)** Traces of altruist fraction for 5 simulations of structured population simulation mimicking population dynamics of gut microbiota across multiple hosts. Parameters for the simulation: $\:A=28,\:B=0.05,\:C=1{0}^{-8},\:{\psi\:}_{0}=1{0}^{8}$ $,\:E=0.0125,\:F=4.0,G=1$ $,\:T=1,\:b=0.4,\:d=0.01,\:K=20,\:{r}_{m}=1.0,\:f=1{0}^{-4}$. For these conditions $\:{a}_{opt}=0.98716,\:{r}_{opt}=0.00584$ ($\:\frac{{r}_{opt}}{E}=0.047$). See Methods for details. **(C)** Traces of subpopulation sizes in an example structured population. Populations containing higher cheater fractions have smaller equilibrium sizes and are less likely to send migrants to colonize new hosts

As expected, simulating competition between a PCD-competent subpopulation employing the optimal defense strategy for the specified (PCD-sensitive) environmental conditions and a PCD-incompetent subpopulation with the same investment in immunity but no investment in PCD, typically results in collapse of the PCD-competent subpopulation (Fig. 5A). Unexpectedly, however, in 20% of the ecologies evaluated, PCD competency was found to be a neutral trait such that the population composition was observed to be stable up to stochastic fluctuations over the course of the simulation (Fig. 5A). The mechanism underlying this behavior may be understood as follows. Committing PCD incurs a fitness cost proportional to the number of progeny the cell would have produced, on average, if it had not committed PCD. When the PCD threshold is high, and PCD is only committed if the intracellular viral load is high, the expected number of progeny is negligible. It follows that in many ecologies, even within unstructured populations, PCD-competency is insensitive to displacement by “cheaters”. See Supplementary Fig. 6 for a visualization of the expected number of progeny lost as a result of committing PCD stratified by cell size and intracellular viral load.

For the remaining majority of ecologies, PCD is evolutionarily unstable within a well-mixed population; however, we demonstrate the locally optimal defense investment strategies predicted by our model are evolutionarily stable in a structured population. Many explanations for the emergence and maintenance of altruistic traits have been proposed [59–62], including with respect to the emergence of PCD specifically [37, 63, 64]. Here we formulate a version of the Simpson’s paradox model of altruism maintenance [42, 43] where each subpopulation is represented by a single chemostat as described in previous sections. Simpson’s paradox assumes an ensemble of subpopulations with limited migration (including, for example, residents of the human gut microbiome which we explicitly discuss in this work or, alternatively, biofilm-forming species). When the subpopulations including altruists grow faster or are larger, which is almost always the case with respect to PCD investment, these altruistic subpopulations are more likely to seed new colonies. Therefore, paradoxically, an altruistic genotype can fix globally even if local subpopulations are prone to the invasion of cheaters. The global maintenance of an altruistic genotype cannot be predicted from its (in)ability to outcompete a cheater genotype locally.

We model the population structure as a fully connected network of $\:k$ chemostats (subpopulations) each containing altruists and cheaters. Migration occurs between the subpopulations at a constant rate $\:m$. Crucially, as per Simpson’s paradox, larger populations are more likely to send out migrants: at the time of migration, the probability that a subpopulation $\:i$ will send out migrants is equal to $\:{P}_{i}={N}_{i}/{\sum\:}_{j=1}^{k}{N}_{j}$ where $\:{N}_{i}$ is the size of *i*^*th*^ population. The destination population is chosen with uniform probability. The number of migrants $\:{n}_{m}$ is then chosen from a binomial distribution with parameters $\:n={N}_{i}\:,\:p=f$ where $\:f$ specifies the probability of migration per individual in the subpopulation. The number of altruists in the migrant pool $\:{n}_{ma}$ is then drawn from the binomial distribution with parameters $\:n={n}_{m},\:p={N}_{ia}/{N}_{i}$ where $\:{N}_{ia}$ is the number of altruists in the subpopulation *i* (it follows that the number of cheaters is $\:{n}_{mc}=\:{n}_{m}-{n}_{ma}$).

Chemostat microenvironments are also subject to a birth-death process with birth and death rates of $\:b\frac{(K-k)}{K}$ and $\:d$, respectively, modelling the population dynamics of a metapopulation with carrying capacity $\:K$. Chemostats are born empty (with subpopulations of size 0) and are colonized through subsequent migration. Among other biological systems, this model approximates the dynamics expected among bacterial symbionts colonizing the guts of an animal host population where each gut is represented by a chemostat and new hosts are born without gut symbionts.

Figure 5B shows the dynamics of PCD fixation across 5 replicate structured metapopulations within a PCD-sensitive ecology (where the PCD-competent subpopulation is outcompeted within a single, isolated well-mixed population, see Supplementary Figure 7). We observed altruist fixation across all simulations confirming that the locally optimal defense investment strategies predicted by our model are evolutionarily stable in structured populations. Figure 5C illustrates the metapopulation dynamics of a single representative simulation.

## Discussion

In this work, we combined mathematical modeling with comparative genomics and metagenome analysis to explore the ecological determinants of prokaryote antiviral defense strategy, and in particular, the choice between active immunity and PCD. Overall, a combined strategy of investment in both PCD and immunity was found to be optimal, consistent with the identification of multiple defense systems in most genomes, with potential for synergistic activity [30, 65] and demonstrated interplay between immunity and PCD [33, 34].

**Fig. 6:**
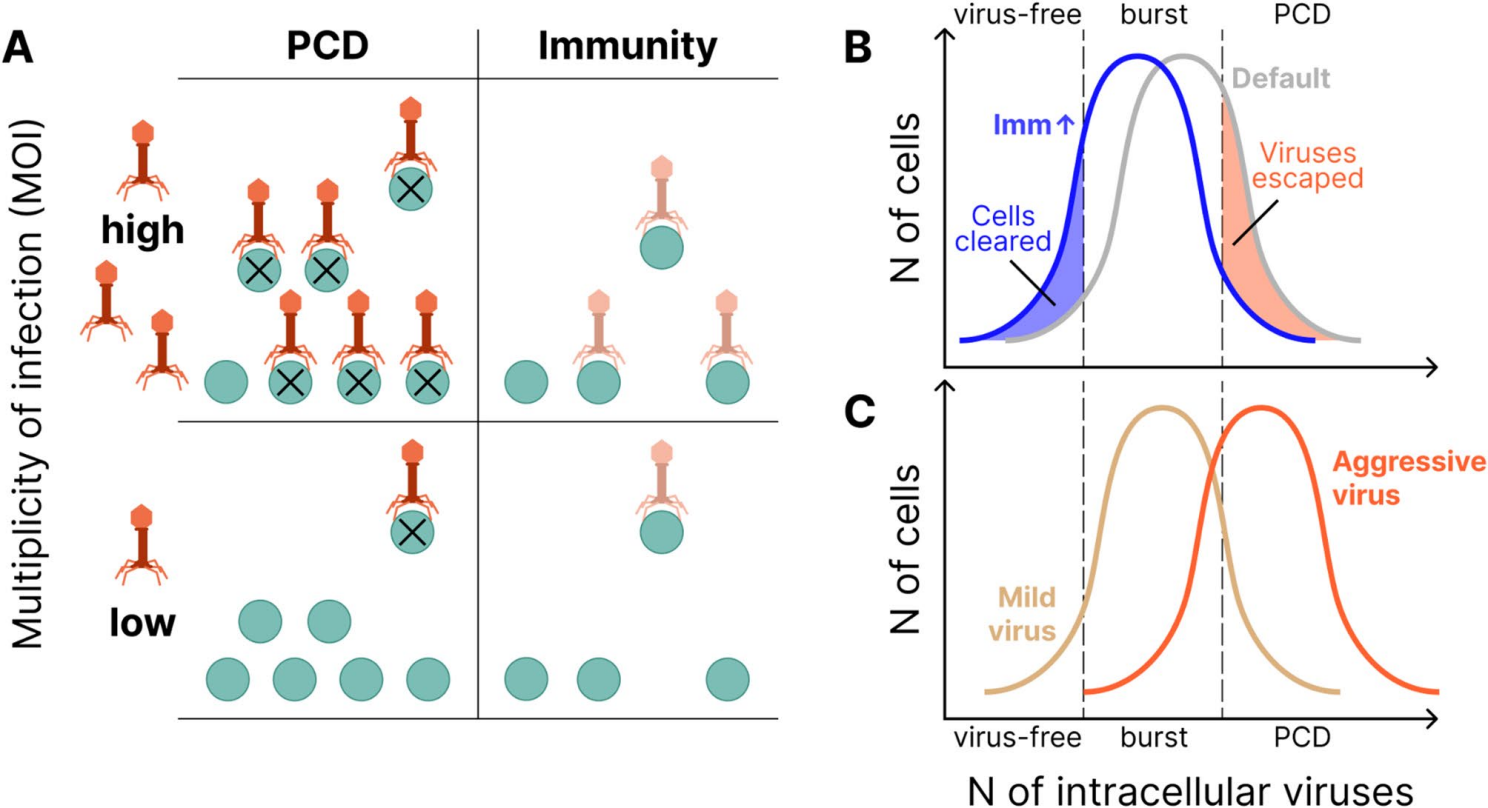
Relationships between active immunity and PCD in prokaryote antivirus defense. **(A)** PCD is advantageous at low extrinsic MOI (i.e. high population size and/or low viral pressure), immunity is advantageous at high extrinsic MOI (i.e. low population size and/or high viral pressure). **(B)** Immunity may directly antagonize PCD by keeping the viral concentration near the burst size but below the PCD threshold. Two idealized distributions of cells within the chemostat are shown where the y-axis specifies the relative proportion of cells with intracellular virus number specified by the x-axis. These distributions are: default and increased Immunity, which shifts the virus load lower. **(C)** (Axes same as in B.) Viruses with high replication rates are more easily cleared by PCD and consequently may result in reduced cell loss relative to more “mild” strains

Our model predicts two principal determinants of optimal defense strategy which were supported by genome analysis, namely, viral pressure and host census population size (Fig. 6A). As viral pressure grows, both the predicted optimal investment and the observed genomic investment in PCD decrease relative to the investment in immunity. It follows that PCD becomes relatively more costly than immunity with increasing viral pressure. Conceivably, under high viral pressure, a large fraction of the cells in the population is infected, so that PCD is likely to cause population collapse due to the lack of a sufficient number of uninfected cells to restore growth.

Conversely, as the host population size increases, both predicted optimal investment and the observed genomic investment in PCD increase relative to the investment in immunity. Increasing absolute investment in PCD in larger populations can be understood to be a consequence of the expected number of infections prevented by a single PCD event growing with the total population size because each infected cell is expected to infect multiple neighbors. Conversely, in smaller populations, the loss of each cell due to PCD represents elimination of a larger fraction of the population, increasing the cost for the population and the likelihood of extinction. With respect to immunity, for the optimal investment strategy, absolute investment in immunity may decrease with increasing population size because the absolute total growth cost, in units of cells per unit time, is greater for larger populations.

Building on our previous work [44], we again make a connection with r and K evolutionary strategies [66]. When the carrying capacity is small, K-strategists, which prioritize offspring quality over quantity, outcompete r-strategists, which prioritize the reverse. Investment in immunity, similar to investment in somatic damage repair in our previous work [44], represents a K-strategy whereas investment in PCD, analogous to asymmetric somatic damage allocation, represents an r-strategy. We demonstrated that the relative contribution of PCD to the optimal strategy over diverse ecologies increases with increasing carrying capacity. By contrast, decreased immunity investment was most pronounced in the model within a smaller subset of ecologies where the cost of immunity was relatively high. This trend was not observed in the empirical data, implying that the cost of immunity in the human gut microenvironment is relatively low (Supplementary Fig. 3).

PCD is typically considered altruistic behavior providing defense at the population level. As expected, in the majority of ecologies studied, direct competition with cells that do not commit PCD in a well-mixed environment leads to the local extinction of the PCD genotype (Fig. 5A). However, within a structured population (a version of the Simpson’s paradox model of altruism maintenance [42, 43]) that reflects realistic prokaryote metapopulations such as biofilms or human gut microbiomes, we demonstrate that the predicted optimal defense strategies are evolutionarily stable (Figs. 5B-C). Unexpectedly, in a sizable minority of ecologies, we find that PCD is a neutral trait under competition such that cells commit PCD only when they have a negligible chance of ever dividing again. In this context, the interpretation of PCD as an altruistic behavior is less clear.

PCD is also often assumed to be a “last-ditch effort”, activated under conditions of extreme stress [54, 67]. Indeed, within the minority of ecologies where PCD is a neutral trait, pushing the boundaries of “altruism”, this characterization agrees with our observations from the individual cell perspective: PCD is only committed by cells with such a high viral burden that the immunity investment required to divide again is much greater than the optimal (average) investment. In the majority of the ecologies, where PCD is decidedly altruistic, however, by definition, the cell committing PCD must have a significant probability of dividing again (at the time when PCD is launched), not clearly reflecting a “last-ditch effort”. From the population level perspective, we show that PCD is most strongly preferred when the overall viral burden is low, not under extreme stress (Fig. 2). More generally, these observations fit into our ongoing theoretical work [68] reevaluating the ecological requirements for the evolution of cooperation which, in brief, suggest cooperative behavior is more robust to cheating than currently assumed.

Motivated by the strong agreement between our model predictions for the optimal defense investment strategy and the observed genomic investment, we further explored the dependence of the optimal strategy on the virus replication rate within the model. We identified a complex effect of the replication rate on the optimal strategy, co-dependent on the extrinsic MOI. For many conditions, investments in PCD and Immunity are antagonistic (Fig. 6B). Increasing investment in immunity leads to two opposing outcomes with respect to host fitness, effectively reducing the viral replication rate while also allowing the virus to “slip under the PCD radar” in cells that are not cleared of the virus. This second outcome demonstrates the potential for antagonism between the defense strategies, whereby investment in immunity decreases PCD efficacy. This antagonism is reinforced by the observation that optimal investment in PCD grows with increasing cost of immunity, suggesting that PCD supplants rather than augments immunity in this context. For this antagonism to occur, the immune response must not be completely effective.

If the immune response is triggered exclusively by late gene products, and late gene products are primarily produced once there are many copies of the viral genome within the cell, the timescale between crossing the intracellular viral threshold and deterministic lysis would be very short, limiting the opportunity for antagonism between immunity and PCD. Furthermore, in the current standard experimental protocol for assessing immune activity, the plated phage plaquing assay, controls lacking the studied immune system succumb to infection at phage concentrations up to 1000 fold lower than those carrying the system. Such a binary landscape - the presence or absence of a highly effective immune system - would not support the antagonistic immune vs. PCD behavior we predict here. We propose 2 possible explanations for this apparent contradiction: (1) both intra- and inter-cellular phage kinetics are likely very different in the bulk environment we model from the plated microenvironment. Unlike in the plated microenvironment, in bulk, cells can be exposed to a low extracellular virus concentration for prolonged periods, conditions more likely to elicit an incomplete immune response. (2) Most experimental assays are designed precisely to identify highly effective immune responses elicited by specific systems predicted to target specific viruses. In natural ecologies, a single immune system can be variably effective at protecting against infection by a diverse range of viruses. Under such conditions, our model for antagonism would be most relevant for generalist immune responses [30, 69].

Related to the previous finding, we observed that, surprisingly, the host population size can be nonmonotonic with respect to the virus replication rate (Supplementary Fig. 8). This effect may be rationalized by considering the optimal virus replication rate (from the virus perspective) given a fixed PCD threshold $\:1-a$. The virus would achieve the highest growth rate by bursting the cell immediately before the concentration $\:\rho\:=1-a$ is reached. It follows that the virus replication rate should be fine-tuned to approach, but not exceed, this critical concentration. Consequently, the virus cannot simply maximize its replication rate (Fig. 6C). This finding is also in accordance with our previous work suggesting that under some conditions, the host population size can increase with the introduction of a virulent pathogen [70].

Within our model, we make two critical assumptions regarding the lysis probability as a function of intracellular virus concentration. First, cell division is arrested in the presence of even a single virion and second, lysis probability increases strictly monotonically with the intracellular virus concentration (in other words, any amount of virus confers a nonzero probability of lysis). These assumptions are in agreement with the apparent consensus in the field [25, 71, 72]. However, because only virus-free cells reproduce, cells with intracellular virus concentration far from the lethal threshold comprise the vast majority of the population in many conditions. Consequently, even though the lysis probability is very low for these cells, the skewed population structure produces a two-peaked burst size distribution with one peak approaching the lethal threshold and the other at low virus counts (Supplementary Fig. 2A).

In the results presented here, the model parameters are chosen to minimize the size of this lower peak in order to admit qualitative agreement with existing empirical data. Nonetheless, this finding clearly deviates from the consensus understanding that lytic bacteriophages tend to produce relatively narrow burst size distributions in a particular environment [39, 47, 48, 73]. Notably, experimental protocols most commonly employed to measure virus burst size are limited to measuring average values over many host cells [40, 74, 75]. Bimodality could not be observed using these approaches. Single-cell burst size has more recently been evaluated in an experimental chemostat system similar to the conditions assumed within our model, and substantial variance in the burst size has been demonstrated [76]. Analogous to prior work on prokaryotic aging and size homeostasis [77, 78], if empirically supported with these single-cell methodologies, our results would indicate that the host population structure under lytic viral pressure could be substantially more complex than previously appreciated.

The principal limitation of this study is the asymmetric representation of PCD and immunity investment within the model whereas estimation of investment in both types of systems in the genomic data analysis is symmetric. Within the model, PCD investment bears no explicit growth cost, whereas investment in immunity is costly. The motivation behind this choice of model parameters is twofold. From first principles, the maintenance of PCD machinery, which comes at an infinite fitness cost for individuals activating the pathway, implies that its energetic cost is low. More specifically, it can be expected that PCD machinery is highly expressed only when the cell is en route to death, incurring no substantial transcriptional or translational costs at other times (although toxin-antitoxin pairs are expressed at some level continuously) [4, 79, 80]. Introducing a more sophisticated measure for estimating genomic investment in PCD and adaptive immunity differently would have required additional free variables which would have substantially complicated the data analysis. However, the observation that PCD genes and systems tend to encompass substantially fewer total nucleotides than immune genes and systems (shorter genes and/or fewer genes per system) appears to support the assumption of the low energetic cost of PCD [28]. Another key assumption implicit in our model and relevant for the inference of the relative cost of genomic investment in immunity vs. PCD is that the gene pools of the available immune and PCD systems are infinite (arbitrarily large). Should the number of available systems in one category be substantially smaller than the other, this would alter the observed distribution of defense systems independently from the cost of maintaining each system.

In conclusion, through mathematical modeling and comparative genomics, we assessed the ecological determinants of the predicted optimal and empirically observed investments in antivirus defense systems among prokaryotes, in particular, active immunity and PCD. Uncovering the factors which shape the evolution of defense systems is central to the pursuit of general theoretical models of host-pathogen coevolution. Furthermore, understanding the ecological and host range of defense systems is important for evaluating candidates for phage therapy [35, 36]. Phage cocktail design is subject to optimization to the full complement of defense systems present in the target genome(s), and we hope that our work can help to support further experimental validation of synergistic or antagonistic relationships between different classes of defense systems, as has been previously done for individual systems [30].

We found that investment in PCD is ubiquitous among optimal strategies, and in more than one third of the ecologies studied, host fitness is highly sensitive to deviations in PCD investment relative to the global optimum. We identified two primary ecological determinants of optimal defense strategy. Increasing viral pressure decreases whereas increasing host census population size increases optimal PCD investment. Together, these findings indicate that, generally, PCD is preferred at low extrinsic MOI and immunity is preferred at high extrinsic MOI. These findings can be rationalized from the population-level selection standpoint: eliminating few cells early in the course of infection, at low extrinsic MOI, could prevent the necessity of activating PCD at a later stage which could lead to population collapse. Under these conditions, decreasing the relative investment in immunity is beneficial due to the lowered energetic costs. In some ecologies, we observed direct antagonism between PCD and immunity such that investment in immunity reduces the efficacy of PCD by keeping viruses with high replication rates near the burst size but below the PCD threshold. Similarly, viruses with high replication rates can paradoxically result in reduced overall cell loss as they are more readily cleared by PCD. These findings bring substantial added complexity to the established landscape of prokaryote antiviral defense, emphasizing the importance of continued effort aimed at the discovery and characterization of defense systems.

## Methods

Please see Supplementary Methods, which includes all the material presented below with additional information, for details.

### Chemostat model: well-mixed, local population

Building on our previous work [44], we adopt a model of a chemostat containing a population of prokaryotes exposed to influent media containing both nutrients and virus. Individual cell state is completely described by volume $\:{p}_{0\:}\:\le\:\:p\:\le\:\:2{p}_{0}$and viral load $\:{q\:\le\:\:Q}_{\:}\:$. It follows that the population is specified by a state matrix of size $\:{p}_{0\:}$x $\:Q$ where $\:n(p,\:q)$represents cell counts. At each time step $\:\varDelta\:t$ the matrix is updated as follows:

$$\eqalign{ & \:n(p,\:q,\:t + \Delta \:t)\: = n(p,\:q,\:t) + [A(1 - \frac{r}{E})*\phi \:* \cr & (n(p - 1,\:q,\:t)*(p - 1) - n(p,\:q,\:t)\:*\:p)\: + \cr} $$

$$\eqalign{ & \: + \:F*(n(p,\:q - 1,\:t)*(q - 1)\: - \:n(p,\:q,\:t)*q)\: \cr & + \:r\:*\:p\:*(n(p,\:q + 1,\:t)\: - \:n(p,\:q,\:t))\: - \cr} $$

$$\:-(B+(\frac{\rho\:}{T-\rho\:}{)}^{G}\left)*n\right(p,\:q,\:t\left)\right]\varDelta\:t$$

where each term represents cell growth, cell death, viral replication, and cell immunity, respectively. Parameters are: baseline growth rate, $\:A$; immunity investment, $\:r$; maximum immunity investment, $\:E$; nutrient concentration, $\phi$; virus replication rate, $\:F$; dilution rate, $\:B$; deterministic lysis threshold, $\:T$; lysis probability scale factor, $\:G$; and $\:\rho\:\equiv\:\frac{p}{q}$. Only virus-free cells ingest virus, providing for superinfection exclusion and division is arrested with a single virus copy but cell growth is not prohibited until division volume is reached.

The number of viruses in the chemostat is given by the following equation:

$$\eqalign{ & \:\psi \:(t + \Delta \:t)\: = \:\psi \:\left( t \right)\:\: + \:[(\psi {\:_0} - \psi \:)B\: - C\psi \: \cr & \sum {{\:_p}} (p*n(p,\:0,\:t)) + \sum {{\:_p}} \sum {{\:_q}} (q*n(p,\:q,\:t)* \cr & {(\frac{{\rho \:}}{{T - \rho \:}})^G})]\Delta \:t\: \cr}$$

where each term represents chemostat dilution, ingestion of viruses by cells, and viral reproduction, respectively. Parameters are: virus concentration in influent media, $\:{\psi\:}_{0}$; and cell media uptake rate, $\:C$. Cells that die of viral infection release all intracellular virus particles into the chemostat.

The change of the nutrient concentration is given by the following equation:

$$\eqalign{ & \:\phi \:(t + \Delta \:t)\: = \:\phi \:\left( t \right)\: + \:[(\phi {\:_0} - \phi \:) \cr & B - C\phi \:\sum {{\:_p}} \sum {{\:_q}} (p*n(p,\:q,\:t))]*\Delta \:t\: \cr} $$

where the first term represents chemostat dilution at a rate $\:B$, the second term represents consumption by cells at a rate $\:C$ per unit of cell volume, and $\:{\phi\:}_{0}$ represents the nutrient concentration in influent media. The scripts used to run the simulations can be accessed at https://github.com/Captain-Blackstone/PCD_vs_Immunity_Simulations.

### Structured population model

Our structured population model consists of a fully connected graph of time-varying number of nodes, $\:k$, where each node is a well-mixed subpopulation behaving as defined above generalized to admit the description of 2 cellular genotypes: altruist (PCD-competent), and cheater (PCD-incompetent), interacting with each other through shared media (including both nutrient, $\phi$, and virus, $\:\psi\:,$ exposure). The defense strategy parameters are ($\:{a}_{opt},\:{r}_{opt}$) and ($\:0,\:{r}_{opt}$) for the altruist and cheater genotypes respectively. Subpopulations undergo a birth-death process with birth rate $\:b\frac{(K-k)}{K}$ and death rate $\:d,\:$where $\:K$ is the carrying capacity of the global environment. Subpopulations are born empty and populate as a result of migration. The time between migrations is drawn from an exponential distribution with mean $\:m$. The source subpopulation is chosen with a probability proportional to the subpopulation size rounded to the nearest whole number, the destination subpopulation is chosen with uniform probability, and when migration occurs, the number of migrants $\:{n}_{m}$ to be transferred to the destination subpopulation from the source population is drawn from the binomial distribution with parameters $\:{N}_{i},\:f$ where $\:f$ is a fixed fraction of the source population.

### Analysis of genomic investment in PCD and immunity

To estimate the investment in PCD and immunity in real bacterial genomes we used a combination of two datasets: PADLOC [52] and Prok2311 [53] annotating 223,572 bacterial genomes belonging to 16,441 species with 239 bacterial defense system assignments. Using expert manual review we classified them into 104 PCD systems, 58 immunity systems, and 76 undefined systems. For each genome, we identified all genes associated with PCD and immunity based on the published information on the functionality of prokaryotic defense systems [9, 54, 55, 81]. Briefly, all defense systems capable of specific recognition and targeting of foreign molecules (typically, nucleic acids), such as CRISPR-Cas, that cause virus clearance without damaging the host cell, were classified as immunity and systems that cause dormancy or cell death, such as toxin-antitoxins, were classified as PCD. The complete list of such assignments is provided in Supplementary Table 1.

Using the NCBI utility datasets we downloaded the metadata and extracted genome sizes for all the PADLOC genomes. Since PADLOC did not contain toxin-antitoxin (TA) systems, which we consider to be an important component of bacterial defense via PCD [4, 9], we used the TA annotations from Prok2311. We randomly sampled 1 representative genome per species for a total of 5121 genomes for downstream analysis. We assume that viral pressure ($\:{\psi\:}_{0}$ parameter in our model) is proportional to total genomic investment in defense, including both PCD and Immunity. Alternatively, genomic investment could be proxied by the total length in nucleotides (not normalized by genome length), the total number of defense genes, or the total number of defense systems. Each of these measures has advantages. We chose to report the genome fraction for two principal reasons. First, selective pressures vary with genome size such that the cost of maintaining accessory genes is not equal among large and small genomes [82]. Second, the statistical fits presented in Figs. 2B-D would require greater model complexity to achieve the same maximum likelihood and consequently would result in a worse overall BIC.

It is important to note that, as new defense systems continue to be identified at a rapid pace, we are likely underpredicting the total number of defense systems in most if not all genomes. We expect, however, that the diversity of defense systems represented in our profiles is high enough that the observed ratio of immunity and PCD systems is not systematically biased at the genus level.

### Microbiome abundance data analysis

We downloaded 8982 samples of healthy adults from curated Metagenomic Data [56]. We used the ncbi-taxonomist python module to extract species names from NCBI IDs used in this resource. For each sample we used ranked abundances of all the species. We excluded species that were found in fewer than 100 samples. We then classified the remaining species into subsets representing high-abundant and low-abundant representatives, discarding intermediate species for which abundance was highly variable across individuals. A species was classified as high-abundant if the 1st quartile of the distribution of its rank abundances across samples was higher than a certain threshold $\:{T}_{a}$ (integer value representing the rank threshold); likewise, a species was classified as low-abundant if the 3rd quartile of the same distribution was lower then $\:{T}_{a}$ (Supplementary Fig. 9). We picked the threshold $\:{T}_{a}$ to maximize the number of species for which genomes were available within our dataset belonging to the smallest of the two (high-abundant, low-abundant) categories. To assess to what extent the effect observed was driven by genome size differences between the groups, we calculated the deviation of PCD investment from the expectation derived from genome size alone. For comparison, profiles of genes belonging to COG pathways taken from https://www.ncbi.nlm.nih.gov/research/cog/pathways/ and 1 set of random profiles of the same size as the number of TA profiles (*n* = 159) were extracted and analyzed following the same protocol>.

## Supplementary Information

Below is the link to the electronic supplementary material.

Supplementary Material 1

Supplementary Material 2

Supplementary Material 3

## Data Availability

No datasets were generated or analysed during the current study.
