# Supplementary material for: Ecological determinants of altruism in prokaryote antivirus defense
Source: Biol Direct. 2025 Dec 20;21:7. doi: 10.1186/s13062-025-00699-8 (PMC12805718; doi:10.1186/s13062-025-00699-8)
Supplement: Supplementary file 1 — Supplementary Material 1 [file 13062_2025_699_MOESM1_ESM.docx]

# Ecological determinants of altruism in prokaryote antivirus defense

# Supplementary Figures

### Supplementary Figure 1: PCD-sensitive landscapes


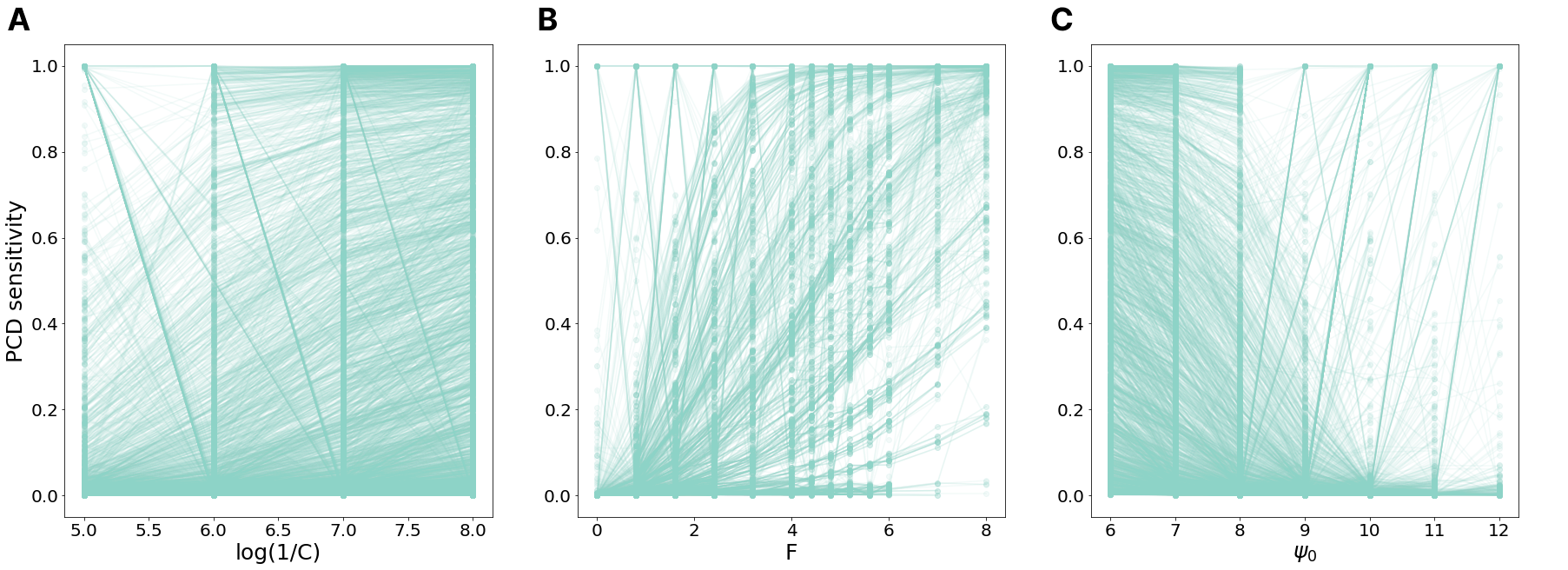
**A**. Fitness landscapes become more sensitive to PCD as population size ($log(1/C)$) increases. **B**. Fitness landscapes become more sensitive to PCD as viral replication rate ($F$) increases. **C**. Fitness landscapes become less sensitive to PCD as viral pressure ($\psi_{0}$) increases.

### Supplementary Figure 2: Burst size parameters


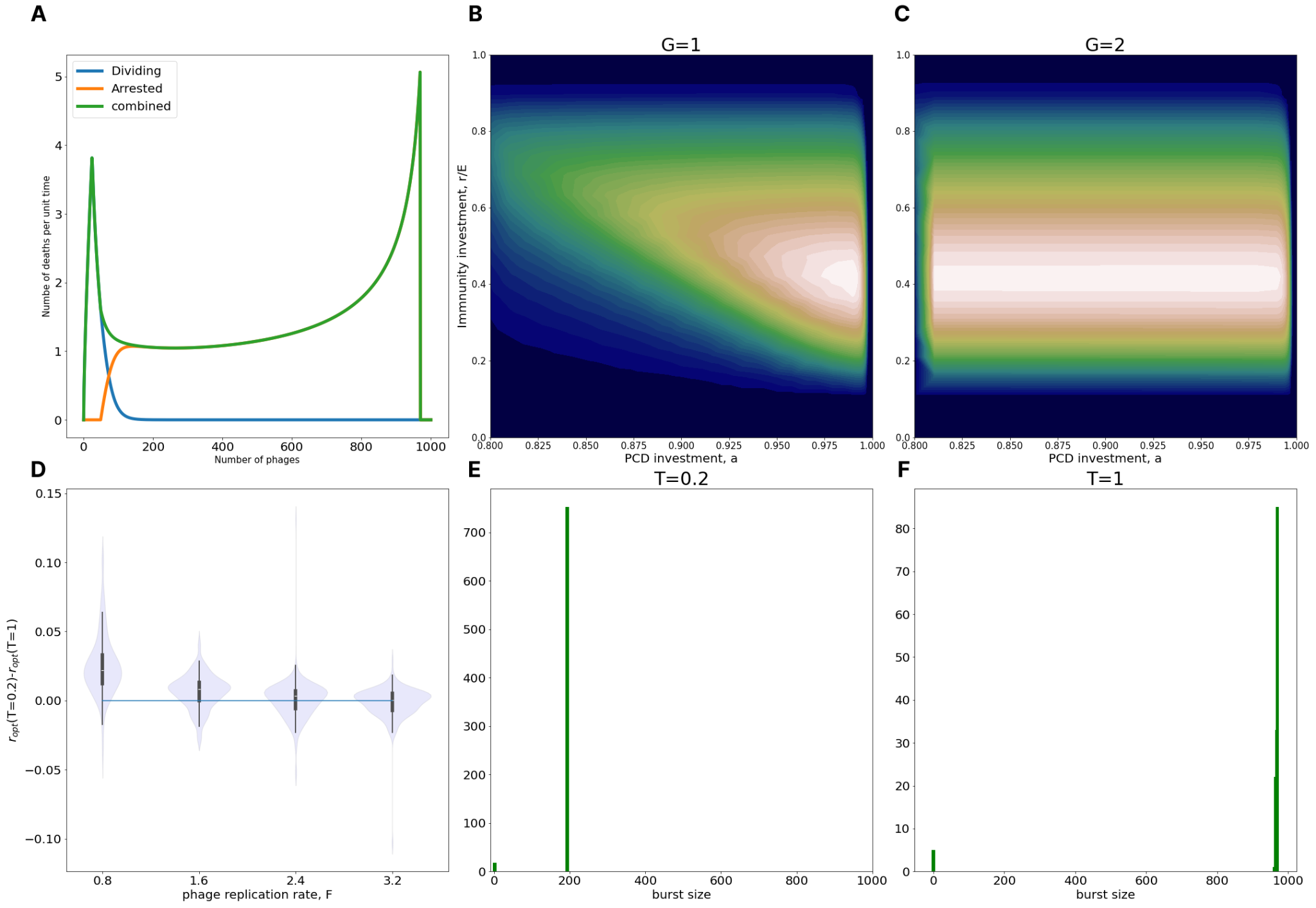


**A**. Equilibrium burst size distribution in an example population ($B=0.25, C=10^{-6}, D=0.0,$ $\psi_{0}=6*10^{6}, E=0.0125, F=1.25, T=1.0, G=0.5$). Burst size distribution is necessarily bimodal: the fact that the majority of the population is infected with few viruses leads to the peak near 0 while the fact that virus-induced death rate diverges as it approaches T leads to the peak at T. **B**. Typical fitness landscape for G=1 **C**. Typical fitness landscape for G=2 (not a PCD-insensitive landscape as defined in the main text, due to very low fitness at $a=0, r=r_{opt}$. **D**. Difference in optimal immunity between $T=1$ and $T=0.2$ for different values of virus replication rate. **E**. Mean burst size of a naive population (a=0, r=0) at $T=0.2$. **F**. Mean burst size of a naive population at $T=1$. E and F collectively show that the $T$ parameter primarily controls the mean burst size of the population and that the burst size distributions in the simulations presented in the main text are always heavily skewed to the $T$ peak.

### Supplementary Figure 3: PCD dependence on 1/C for high E


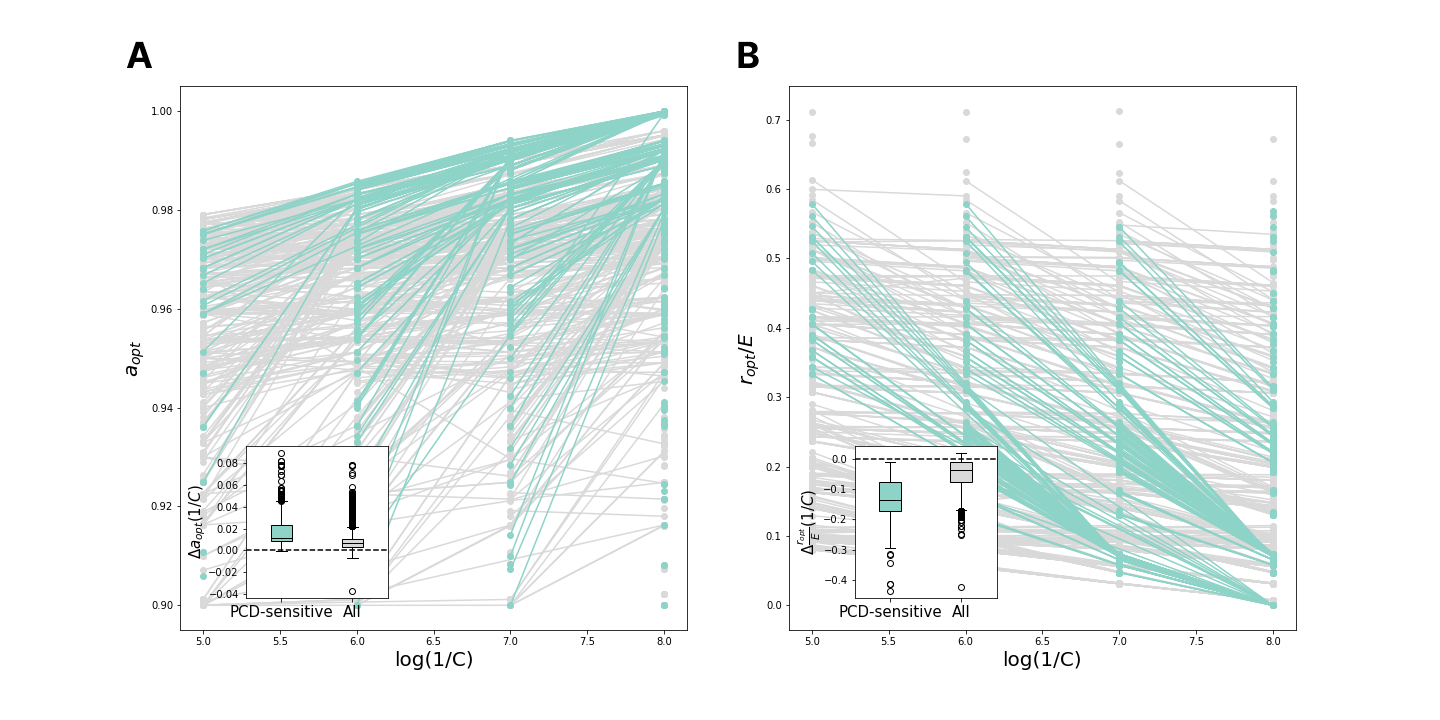
**A**. For high values of E (E=0.05, cheap repair) the distribution of differences in PCD investment between C-neighboring environments is the same as for low values of E (see the Figure 2 of main text). **B**. On the other hand, the differences in immunity investment between C-neighboring environments become smaller for high E. This indicates that for extremely cheap immunity we can observe no difference in immunity investment between high- and low-abundant species, but still observe the difference in PCD investment, compliant with the metagenomic data analysed in the main text.

### Supplementary Figure 4: genome size comparisons


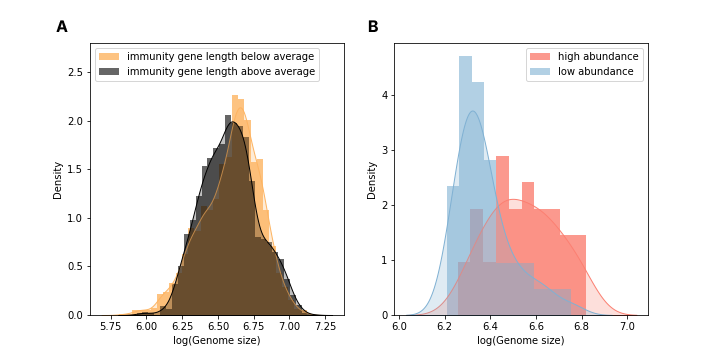


**A**. Distribution of genome sizes between bacteria with high content of immune genes (measured by their combined length in nucleotides) and low content. **B**. Distribution of genome sizes in high-abundance and low-abundance species.

### Supplementary Figure 5: investment in pathways


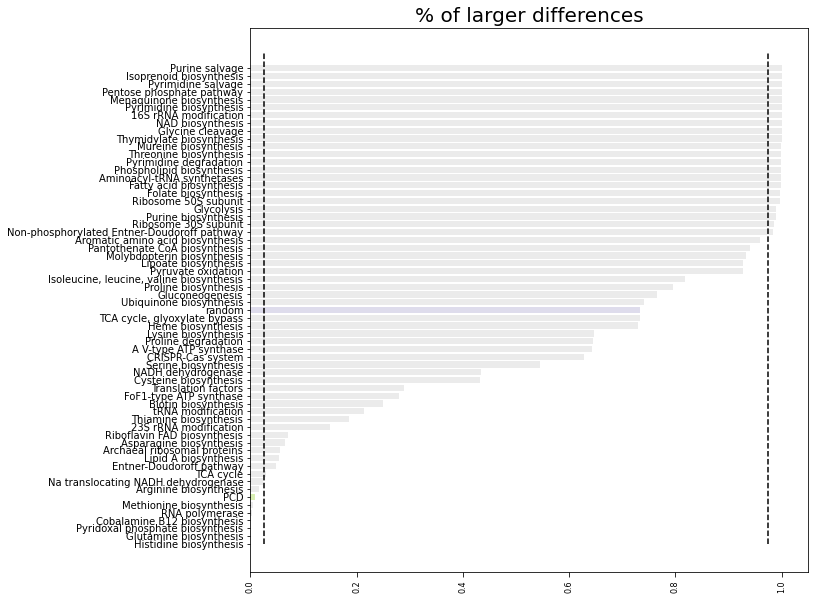


Investment in each system was simulated for both high- and low-abundant species according to the respective genome size distributions (see Methods). The plot shows the % of the simulations that produced larger differences in investment in each system. Random proteins show no deviation from genome-size expectation (blue bar) whereas PCD systems are among those with highest deviations from expectation (green bar).

###

### Supplementary Figure 6: PCD as a neutral trait


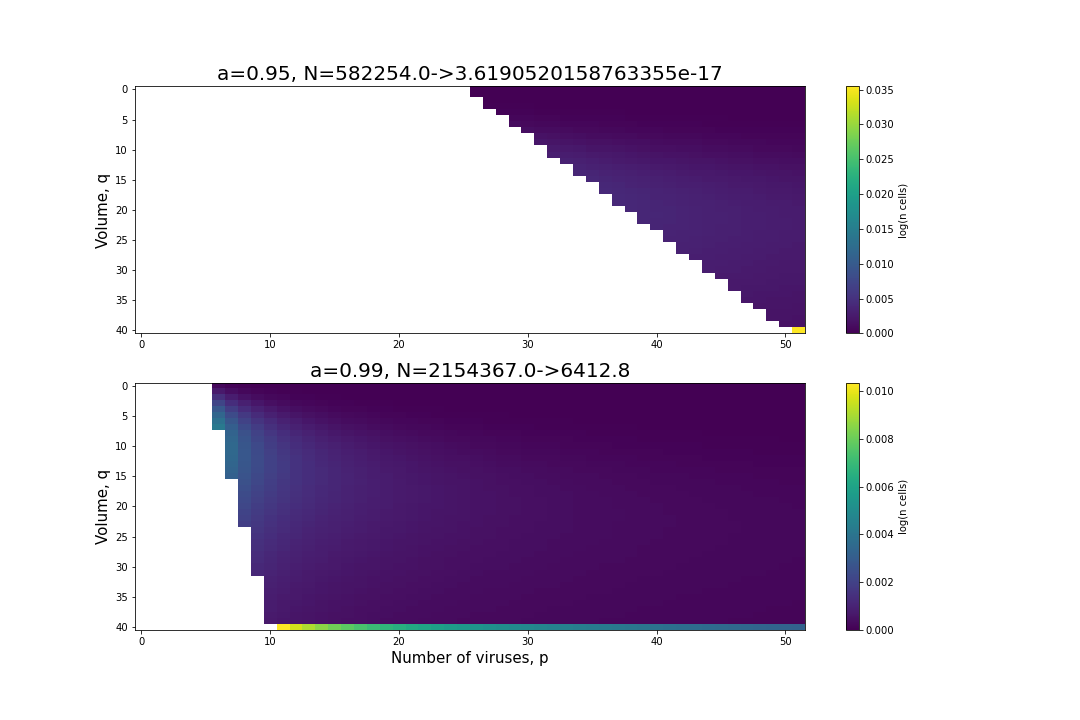


An equilibrium distribution of states $p, q$ was obtained through simulation for $a=0.00, 0.95, 0.99$ (simulations 1, 2, 3 respectively, increasing PCD investment from 0) for a specific environmental condition $A=28.0, B=0.05, C=10^{-8}, \psi_{0}=10^{8}, E=0.0125, F=2.4, H=1.0, G=1.0, r=r_{opt}= 0.004605.$ The top panel shows the difference in distribution between simulation 1 and simulation 2, the bottom panel shows the difference between simulation 1 and simulation 3. If follows that these distributions display the total number of cells which would have divided at least once had they not committed PCD when determined per the environmental conditions and nonzero PCD investment. In the top panel, so few cells may be expected to divide, the result is within the range of numerical error. Consequently, for a=0.95 (PCD is committed at p/q = 0.05) PCD is a neutral trait. In the bottom panel, a modest but significant number of cells (approximately 0.3% of the population) had division events inhibited as a result of PCD activation. Consequently, for a=0.99 (PCD is committed at p/q = 0.01) PCD is an altruistic trait.

### Supplementary Figure 7: Cheater fixation dynamics in a competition experiment


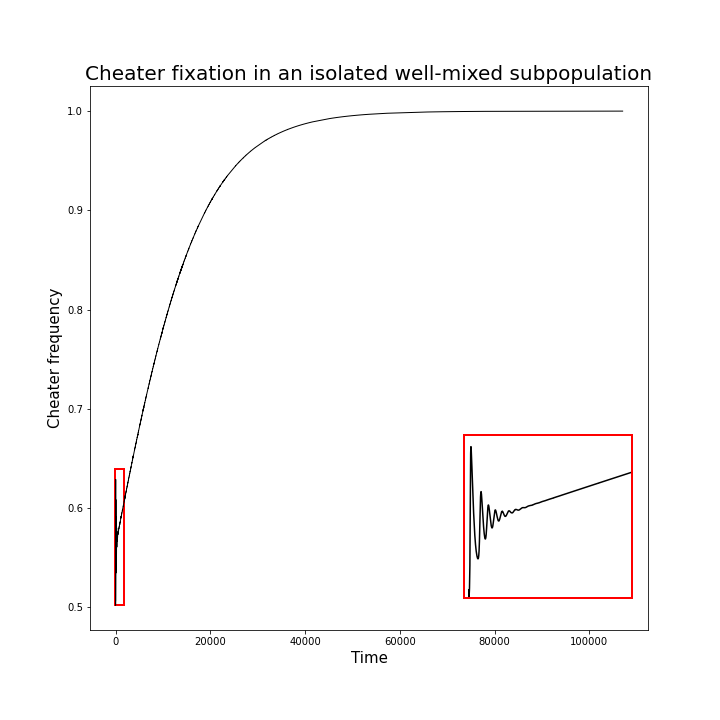


Cheaters and altruists have the same population size and state distribution with respect to cell size and number of viruses ($p, q$) at the start of the simulation.

### Supplementary Figure 8: nonmonotonic population size with F


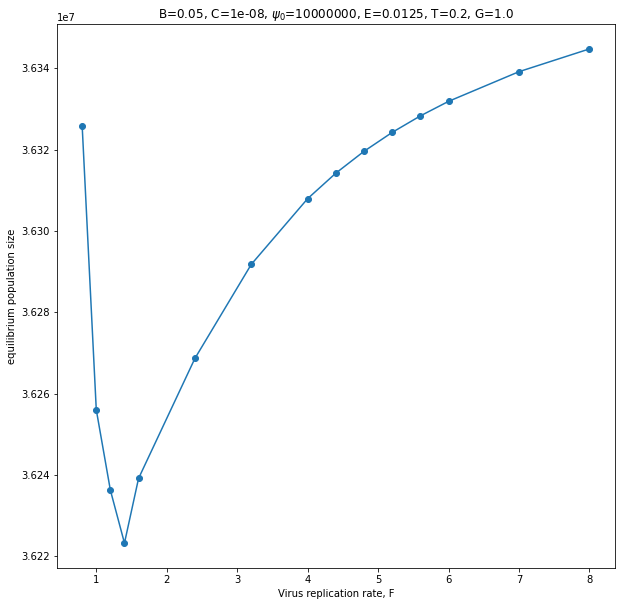


### As the virus replication rate increases, a certain strategy (here a=0.99, r=0.0125) can exhibit nonmonotonic success. At first, increasing viral replication rate decreases the fitness of the population. However, after some threshold, increasing viral replication rate further increases fitness, demonstrating PCD machinery efficiently eliminates against fast-replicating viruses.

### Supplementary Figure 9: low/high abundance classification


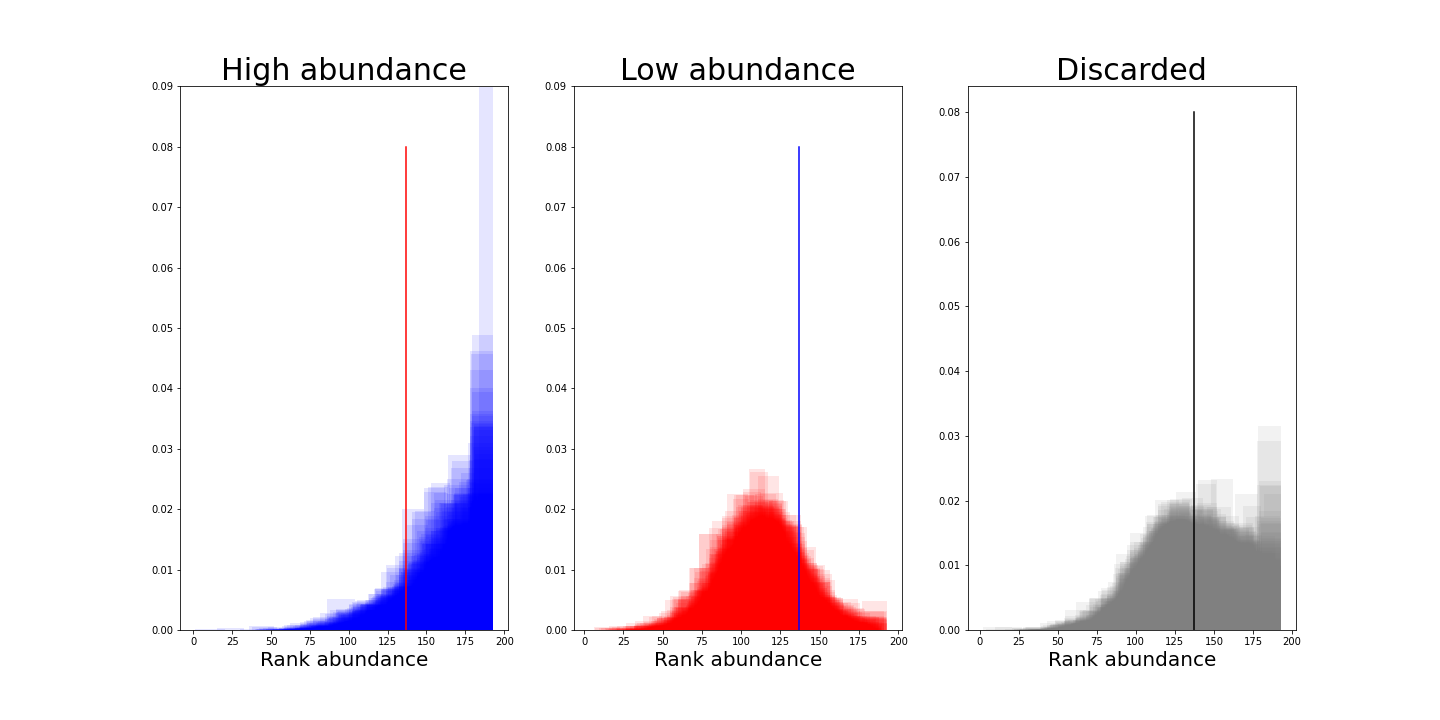


Each distribution represents a single species rank abundance across individual human guts. The vertical lines represent the threshold $T_{a}$ we used to distinguish between high- and low-abundant species (see Methods).
