# Supplementary material for: Ecological determinants of altruism in prokaryote antivirus defense
Source: Biol Direct. 2025 Dec 20;21:7. doi: 10.1186/s13062-025-00699-8 (PMC12805718; doi:10.1186/s13062-025-00699-8)
Supplement: Supplementary file 2 — Supplementary Material 2 [file 13062_2025_699_MOESM2_ESM.docx]

# Ecological determinants of altruism in prokaryote antivirus defense

# Supplementary Methods

## Chemostat model: well-mixed, local population

Building on our previous work^1^, we adopt a model of a chemostat containing a population of prokaryotes exposed to influent media containing both nutrients and virus. Individual cell state is completely described by volume $p_{0} \leq p \leq2p_{0}$and viral load ${q \leq Q}$. It follows that the population is specified by a state matrix of size $p_{0}$x $Q$ where $n(p, q)$represents cell counts. At each time step $\Delta t$ the matrix is updated as follows:

$$n(p, q, t+\Delta t) =n(p, q, t)+[A(1-\frac{r}{E})*\phi*(n(p-1, q, t)*(p-1)-n(p, q, t) * p) +$$

$$+ F*(n(p, q-1, t)*(q-1) - n(p, q, t)*q) + r * p *(n(p, q+1, t) - n(p, q, t)) -$$

$$-(B+(\frac{\rho}{T-\rho})^{G})*n(p, q, t)]\Delta t$$

where each term represents cell growth, cell death, viral replication, and cell immunity, respectively.

1. Cell growth. Cells grow at a baseline rate $A$, and the growth rate is proportional to the nutrient concentration, $\phi$. Investment in immunity, $r$, is growth costly by a factor $1-\frac{r}{E}$. The growth rate is 0 when $r=E$, $E$ representing the maximum possible investment in immunity. When cells reach the critical size $2p_{0}$ they divide provided they contain no virus. We consider division to be arrested with a single virus copy but cell growth is not prohibited until division volume is reached.
2. Cell death. Cells die from chemostat dilution at a rate $B$, from viral infection, and from PCD. The death rate from viral infection increases proportionally with intracellular viral concentration, $\rho=\frac{p}{q}$. This function has two parameters - $T$, the viral concentration at which the lysis probability is 1 and $G$, the slope of the lysis probability function (gradual for small $G$, stepwise for high $G$ . Finally, if the viral concentration $\rho$ is greater than the threshold $1-a$, $a$ representing PCD investment, the cell commits PCD.
3. Viral ingestion and replication. Cells ingest virus from the environment at a rate $C$ proportional to their volume $u$. Only virus-free cells ingest virus, providing for superinfection exclusion. The virus replicates at an exponential rate $F$ proportional to the current number of viruses $q$.
4. Immunity. Cells destroy intracellular viral particles at rate $r$. We assume that larger cells produce more immune proteins, therefore the rate of immune clearance is also proportional to the cell volume, $p$.

The number of viruses in the chemostat is given by the following equation:

$\psi(t+\Delta t) = \psi(t) + [(\psi_{0}-\psi)B -C\psi\sum_{p} (p*n(p, 0, t))+\sum_{p} \sum_{q} (q*n(p, q, t)*(\frac{\rho}{T-\rho})^{G})]\Delta t$

where each term represents chemostat dilution, ingestion of viruses by cells, and viral reproduction, respectively.

1. Ingestion. Cells ingest virus at a rate$C$ per unit of volume,$p$.
2. Viral reproduction. Cells that die of viral infection release all intracellular virus particles into the chemostat.
3. Chemostat dilution. A proportion$B$ of the chemostat volume is removed per unit time, in turn removing a proportion $B$ of the extracellular virus and cells. The same process introduces new virus into the chemostat. $B\psi_{0}$ viruses are introduced per unit time.

Finally, the change of the nutrient concentration is given by the following equation:

$$\phi(t+\Delta t) = \phi(t) + [(\phi_{0}-\phi)B-C\phi\sum_{q} \sum_{p} (p*n(p, q, t)) ]*\Delta t$$

where the first term represents chemostat dilution at a rate $B$ and the second term - consumption by cells at a rate $C$ per unit of cell volume.

We explore different environmental conditions specified by parameters $B, C, E, F, T, G$ and $\psi_{0}$. For each environment, we reconstruct the fitness landscapes with respect to defense investment strategy by numerically solving the system of equations until the equilibrium state is reached for a range of $1-T\leq a \leq1$ and $0 \leq r \leq E$. The fitness of an investment phenotype $a_{0}, r_{0}$ in a given environment is defined as the equilibrium size of the cell population adopting this strategy (or the geometric mean of peaks if the population size exhibited cyclic behavior at pseudo steady state).

The scripts used to run the simulations can be accessed at <https://github.com/Captain-Blackstone/PCD_vs_Immunity_Simulations>.

## Structured population model

Our structured population model consists of a fully connected graph of time-varying number of nodes, $k$, where each node is a well-mixed subpopulation behaving as defined above generalized to admit the description of 2 cellular genotypes: altruist (PCD-competent), and cheater (PCD-incompetent), interacting with each other through shared media (including both nutrient, $\phi$, and virus, $\psi,$ exposure). The defense strategy parameters are ($a_{opt}, r_{opt}$) and ($0, r_{opt}$) for the altruist and cheater genotypes respectively.

Subpopulations undergo a birth-death process with birth rate $b\frac{(K-k)}{K}$ and death rate $d,$where $K$ is the carrying capacity of the global environment (for example the human host population in the context of gut symbionts). Subpopulations are born empty and populate as a result of migration. The initial conditions in all newborn subpopulations are fixed ($\phi=\phi_{0}, \psi=\psi_{0}).$ The time between migrations is drawn from an exponential distribution with mean $m$. The source subpopulation is chosen with a probability proportional to the subpopulation size rounded to the nearest whole number, $N_{i}$: $P_{i}=N_{i}/\sum_{j=1}^{k} N_{j}$ and the destination subpopulation is chosen with uniform probability. When migration occurs, the number of migrants $n_{m}$ to be transferred to the destination subpopulation from the source population is drawn from the binomial distribution with parameters $N_{i}, f$ where $f$ is a fixed fraction of the source population. Across all simulations, $f=10^{-4}$ (typically dozens of cells are chosen for migration). The number of migrants with altruist genotype $n_{ma}$ is drawn from a binomial distribution with parameters $n_{m},{N_{i}}^{a}/N_{i}$ where ${N_{i}}^{a}$ is the number of altruists in the source subpopulation (consequently, the number of migrating cheaters is $n_{mc}=n_{m}-n_{ma}$). Migration probability is independent of the volume and virus load state, ($p, q$).

## Analysis of genomic investment in PCD and Immunity

To estimate the investment in PCD and immunity in real bacterial genomes we used a combination of two datasets: PADLOC^2^ and Prok2311^3^. We downloaded the tables of identified defense genes with defense system assignments for 223572 bacterial genomes belonging to 16441 species from PADLOC. This dataset contained a total of 239 bacterial defense systems. Using expert manual review we classified them into 104 PCD systems, 58 immunity systems, and 76 undefined systems. For each genome, we identified all genes associated with PCD and immunity based on the published information on the functionality of prokaryotic defense systems^4^[^,5,6,7^](https://www.zotero.org/google-docs/?HHkNlX). Briefly, all defense systems capable of specific recognition and targeting of foreign molecules (typically, nucleic acids), such as CRISPR-Cas, Restriction-Modification, BREX, DISARM, DNA phosphorothioation, and others, were classified as immunity, that is, mechanisms that cause virus clearance without damaging the host cell. By contrast, systems that cause dormancy or cell death, such as various abortive infection modules, toxin-antitoxins, CBASS, and others, were classified as PCD. The complete list of such assignments is provided in Supplementary Table 1.

We disregarded the systems marked as “PDC” (Phage Defense Candidate) in PADLOC as we could not assign labels to them, and, moreover, observed them to often be false positives. We then counted the number of genes belonging to PCD and Immunity systems in each genome and recorded the combined length in nucleotides of these two groups of genes. Alternatively, genomic investment could be proxied by the total length in nucleotides (not normalized by genome length), the total number of defense genes, or the total number of defense systems. Each of these measures has advantages. We chose to report the genome fraction for two principal reasons. First, selective pressures vary with genome size such that the cost of maintaining accessory genes is not equal among large and small genomes^8^. Second, the statistical fits presented in Figures 2B-D would require greater model complexity to achieve the same maximum likelihood and consequently would result in a worse overall BIC.

It is important to note that, as new defense systems continue to be identified at a rapid pace, we are likely underpredicting the total number of defense systems in most if not all genomes. We expect, however, that the diversity of defense systems represented in our profiles is high enough that the observed ratio of immunity and PCD systems is not systematically biased at the genus level.

Using the NCBI utility datasets^9^ we downloaded the metadata and extracted genome sizes for all the PADLOC genomes. Since PADLOC did not contain toxin-antitoxin (TA) systems, which we consider to be an important component of bacterial defense via PCD^10^[^,4^](https://www.zotero.org/google-docs/?HYZxGq), we used the TA annotations from Prok2311. We extracted TA genes - those containing either a toxin or an antitoxin profile, and not containing any other functional profiles. We retained only pairs of toxins-antitoxins. A toxin and an antitoxin were considered a pair if each of them was the closest toxin/antitoxin to the other, and if there were no other genes located between them. The intersection between PADLOC and Prok2311 consisted of 25934 genomes belonging to 5121 species. We then randomly sampled 1 representative genome per species for a total of 5121 genomes.

We assume that viral pressure ($\psi_{0}$ parameter in our model) is proportional to total genomic investment in defense, including both PCD and Immunity. We fit a lognormal model of the form $I_{PCD} = \beta*{I_{Imm}}^{\alpha}$ where $I_{PCD}$ and $I_{Imm}$ are genomic investments in PCD and Immunity respectively and $\alpha$ and $\beta$ are free parameters. More precisely, we fitted $\alpha, \beta$ and the noise parameter $\epsilon$ were selected to maximize the likelihood function:

$$logL= \sum_{I_{Imm}, I_{PCD}} log(logNorm.pdf(I_{Imm}, I_{best}, \epsilon) +log(logNorm.pdf(I_{PCD}, \beta*{I_{best}}^{\alpha}, \epsilon))$$

This function assumes that for the true optimal investment in immunity, $I_{best}$, the observed value $I_{Imm}$ is drawn from a distribution centered on $I_{best}$ with lognormally distributed noise $\epsilon$; the observed value $I_{PCD}$ is drawn from a distribution centered on $\beta*{I_{best}}^{\alpha}$ with the same $\epsilon$ noise term. The value $I_{best}$ was chosen for each point $I_{Imm}, I_{PCD}$ separately maximizing the total likelihood. We dropped 464 genomes with $I_{Imm}=0$ or $I_{PCD}=0$ which could not be suitably accommodated within our likelihood function.

## Microbiome abundance data analysis

We downloaded 8982 samples of healthy adults from curatedMetagenomicData^1^[^1^](https://www.zotero.org/google-docs/?QvvlGG). We used the ncbi-taxonomist python module to extract species names from NCBI IDs used in this resource. For each sample we used ranked abundances of all the species. We excluded species that were found in fewer than 100 samples. We then classified the remaining species into subsets representing high-abundant and low-abundant representatives, discarding intermediate species for which abundance was highly variable across individuals. A species was classified as high-abundant if the 1st quartile of the distribution of its rank abundances across samples was higher than a certain threshold $T_{a}$ (integer value representing the rank threshold); likewise, a species was classified as low-abundant if the 3d quartile of the same distribution was lower then $T_{a}$ (Supplementary Figure 9). We picked the threshold $T_{a}$ to maximize the number of species for which genomes were available within our dataset belonging to the smallest of the two (high-abundant, low-abundant) categories. This procedure yielded 39 high-abundant and 37 low-abundant species. We used the Mann-Whitney U test to determine statistical significance between these two groups with respect to genomic investment in PCD and Immunity.

To assess to what extent the effect observed was driven by genome size differences between the groups, we calculated the deviation of PCD investment from the expectation derived from genome size alone. We used Markov Chain Monte Carlo to fit a bayesian linear regression (with intercept, slope and gaussian noise terms) for PCD investment vs genome size on all the 5121 species within our dataset. We then simulated 10000 ensembles of PCD investments corresponding to genome sizes of high- and low-abundant species and obtained the distribution of simulated means. The fraction of simulated means that was greater (or less) than the empirically observed mean investment was interpreted as a p-value. Similarly we obtained the distribution of differences between the simulated means of PCD investments for high- and low-abundant species and calculated the p-value for the empirically observed difference. This procedure was repeated for immunity investment.

For comparison, profiles of genes belonging to 61 COG pathways (excluding only PhotosystemII) (taken from <https://www.ncbi.nlm.nih.gov/research/cog/pathways/>) and 1 set of random profiles of the same size as the number of TA profiles (n=159) were extracted and analyzed following the same protocol.

# References

1[. Biba, D. A., Wolf, Y. I., Koonin, E. V. & Rochman, N. D. Balance between asymmetric allocation and repair of somatic damage in unicellular life forms as an ancient form of r/K selection. *Proc. Natl. Acad. Sci.* **121**, e2400008121 (2024).](https://www.zotero.org/google-docs/?gXFHEG)

2[. Payne, L. J. *et al.* PADLOC: a web server for the identification of antiviral defence systems in microbial genomes. *Nucleic Acids Res.* **50**, W541–W550 (2022).](https://www.zotero.org/google-docs/?gXFHEG)

3[. Galperin, M. Y. *et al.* COG database update 2024. *Nucleic Acids Res.* **53**, D356–D363 (2025).](https://www.zotero.org/google-docs/?gXFHEG)

4[. Georjon, H. & Bernheim, A. The highly diverse antiphage defence systems of bacteria. *Nat. Rev. Microbiol.* **21**, 686–700 (2023).](https://www.zotero.org/google-docs/?gXFHEG)

5[. Lopatina, A., Tal, N. & Sorek, R. Abortive Infection: Bacterial Suicide as an Antiviral Immune Strategy. *Annu. Rev. Virol.* **7**, 371–384 (2020).](https://www.zotero.org/google-docs/?gXFHEG)

6[. Bernheim, A. & Sorek, R. The pan-immune system of bacteria: antiviral defence as a community resource. *Nat. Rev. Microbiol.* **18**, 113–119 (2020).](https://www.zotero.org/google-docs/?gXFHEG)

[7. Makarova, K. S., Wolf, Y. I. & Koonin, E. V. Defense against viruses and other genetic parasites in prokaryotes. (2021).](https://www.zotero.org/google-docs/?gXFHEG)

8[. Lynch, M. & Conery, J. S. The Origins of Genome Complexity. *Science* **302**, 1401–1404 (2003).](https://www.zotero.org/google-docs/?gXFHEG)

9[. O’Leary, N. A. *et al.* Exploring and retrieving sequence and metadata for species across the tree of life with NCBI Datasets. *Sci. Data* **11**, 732 (2024).](https://www.zotero.org/google-docs/?gXFHEG)

10[. LeRoux, M. & Laub, M. T. Toxin-Antitoxin Systems as Phage Defense Elements. *Annu. Rev. Microbiol.* **76**, 21–43 (2022).](https://www.zotero.org/google-docs/?gXFHEG)

11[. Pasolli, E. *et al.* Accessible, curated metagenomic data through ExperimentHub. *Nat. Methods* **14**, 1023–1024 (2017).](https://www.zotero.org/google-docs/?gXFHEG)
